# Anatomically grounded estimation of hindlimb muscle sizes in Archosauria

**DOI:** 10.1111/joa.13767

**Published:** 2022-10-07

**Authors:** Andrew R. Cuff, Ashleigh L. A. Wiseman, Peter J. Bishop, Krijn B. Michel, Raphäelle Gaignet, John R. Hutchinson

**Affiliations:** ^1^ Structure and Motion Laboratory, Department of Comparative Biomedical Sciences Royal Veterinary College Hatfield UK; ^2^ Human Anatomy Resource Centre University of Liverpool Liverpool UK; ^3^ Museum of Comparative Zoology and Department of Organismic and Evolutionary Biology Harvard University Cambridge USA; ^4^ Geosciences Program Queensland Museum Brisbane Queensland Australia

**Keywords:** comparative anatomy, muscle force, myology, palaeontology, physiological cross‐sectional area

## Abstract

In vertebrates, active movement is driven by muscle forces acting on bones, either directly or through tendinous insertions. There has been much debate over how muscle size and force are reflected by the muscular attachment areas (AAs). Here we investigate the relationship between the physiological cross‐sectional area (PCSA), a proxy for the force production of the muscle, and the AA of hindlimb muscles in Nile crocodiles and five bird species. The limbs were held in a fixed position whilst blunt dissection was carried out to isolate the individual muscles. AAs were digitised using a point digitiser, before the muscle was removed from the bone. Muscles were then further dissected and fibre architecture was measured, and PCSA calculated. The raw measures, as well as the ratio of PCSA to AA, were studied and compared for intra‐observer error as well as intra‐ and interspecies differences. We found large variations in the ratio between AAs and PCSA both within and across species, but muscle fascicle lengths are conserved within individual species, whether this was Nile crocodiles or tinamou. Whilst a discriminant analysis was able to separate crocodylian and avian muscle data, the ratios for AA to cross‐sectional area for all species and most muscles can be represented by a single equation. The remaining muscles have specific equations to represent their scaling, but equations often have a relatively high success at predicting the ratio of muscle AA to PCSA. We then digitised the muscle AAs of *Coelophysis bauri*, a dinosaur, to estimate the PCSAs and therefore maximal isometric muscle forces. The results are somewhat consistent with other methods for estimating force production, and suggest that, at least for some archosaurian muscles, that it is possible to use muscle AA to estimate muscle sizes. This method is complementary to other methods such as digital volumetric modelling.

## Graphical abstract

Digitised muscle attachment areas from the hindlimbs of Nile crocodiles, tinamous and four other birds were used to estimate muscle physiological cross‐sectional areas. Some significant relationships were found, so it was deemed justifiable to estimate these areas in the theropod dinosaur *Coelophysis*, obtaining some different values from other studies'. This method is complementary to others such as volumetric 3D reconstruction.

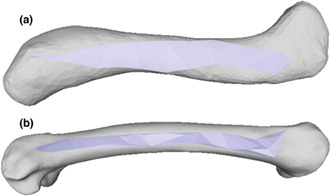

## 1 INTRODUCTION

Animal locomotion is driven largely by active muscular force production (e.g. Alexander, 1992; Biewener, 2003). The maximal isometric force that these skeletal muscles can generate is directly proportional to the physiological‐cross‐sectional area (PCSA) of the muscles (e.g. Medler, 2002; Michel et al., 2020; Powell et al., 1984). Hence information on PCSAs of muscles, and other ‘muscle architecture’ data such as muscle fibre or fascicle lengths and pennation angles, are valuable for relating form to function in the musculoskeletal system (for reviews see Bishop, Wright, et al., 2021; Martin et al., 2020). In extant and extinct taxa alike, there are strong indications from skeletal macro‐ and microstructure where many tendinous, ‘fleshy’, aponeurotic and fibrocartilaginous attachments (e.g. ‘entheses’) are on bone surfaces (Benjamin et al., 1995; Bryant & Seymour, 1990; Suzuki et al., 2002; Whitebone et al., 2021; Woo et al., 1988).

A maxim that is at least implicit in many anatomical studies of extant and extinct tetrapods is that the attachment areas (AAs) of muscles (e.g. their origins) on bones or other tissues allow inferences about the PCSAs of the muscles themselves, at least approximately (Bryant & Seymour, 1990). Theoretically, this maxim stems from the widely held concept that bone models in response to muscular loads, so geometry such as muscle‐bone AAs, cortical bone cross‐sectional area or thickness underlying muscle (Kikuchi et al., 2012; Slizewski et al., 2013) or overall bone shape should correlate with musculoskeletal biomechanics and morphology (e.g. Cornette et al., 2015). The correlation of PCSAs with AAs has been demonstrated with varied success for the jaw muscles of some vertebrates (e.g. Antón, 2000; Bates et al., 2021; Broyde et al., 2021; Thomason, 1991; Toro‐Ibacache et al., 2015). Rabey et al. (2015), however, warned that while experimental alteration of locomotor activity in mice did cause increased limb muscle PCSAs, muscle AAs (the deltoid crest enthesis) did not respond to activity. In contrast, Deymier‐Black et al. (2015) found significant, near‐isometric scaling correlations between supraspinatus PCSAs and tendon AAs across five species of mammals; albeit with wide confidence intervals at extremes (see their figure 4); hinting at conservative interspecific relationships that might relate to maintenance of constant stresses. Theory and empirical data thus may sometimes seem at odds.

Most applicable data on how PCSAs correlate with AAs come from mammalian skulls and jaw muscles. Thomason (1991: his figure 3) found strong correlation between carnivoran jaw muscle PCSAs used to estimate forces, and the projected area of the temporal fossa—a proxy for AA widely used in the ‘dry skull method’. Law and Mehta (2019) also uncovered similar correlations for sea otters using the dry skull method, although they cautioned that different muscles have rather different correlations, and sexual dimorphism as well as ontogeny complicate these correlations. Antón (2000), however, found that macaque species have varying allometries of pterygoid jaw muscles and overall noisy correlations between PCSAs and AA (or origin‐insertion distances: see their figure 4), expressing scepticism that PCSA estimation from AA could reliably be conducted with fossil primates. Toro‐Ibacache et al. (2015) supported this concern, showing divergent relationships between PCSAs and AAs in temporalis versus masseter muscles of humans, and overall poor correlations. Analogously, Davis et al. (2010) found with a three‐dimensional analysis of jaw muscles in bats that PCSAs were sometimes underestimated, sometimes overestimated (consistently for certain muscles), yet regardless bite force could be estimated fairly well. For limb (hand) muscles, Williams‐Hatala et al. (2016) discovered no significant correlation between opponens muscle PCSAs or other architectural variables and tendon enthesis AAs in humans, despite good repeatability of the method. Together, available data hint that PCSA:AA relationships may vary widely across muscles, individuals and taxa. Hence empirical data are essential to test theoretical assumptions of a strong PCSA:AA correlation, on case‐by‐case bases. Some studies have partly circumvented this problem by scaling PCSAs from AAs or via the dry skull method; e.g., fossil *Smilodon* jaw and neck muscle reconstructions using *Felis* data in McHenry et al. (2007).

In two related recent studies of the jaw muscles of rodents, Broyde et al. (2021) and Bates et al. (2021) inferred that estimations of muscle PCSA (and other aspects of architecture) from skull geometry using volumetric modelling were unreliably imprecise, so palaeontological estimates might be particularly fraught with imprecision. Broyde et al.'s (2021) imprecision was generally driven by one investigator (their #3) with little hands‐on experience in myology, although the two other investigators involved also had important sources of error. The most experienced investigator (their #2) broadly tended to produce the best estimates of muscle architecture. None of the investigators had hands‐on experience with the anatomy of the study taxa, so the results can be viewed as somewhat of a ‘worst case’ analogous to studies of fossils. These errors were rooted in the subjectivity of myological reconstruction but also investigator knowledge of basic myology. Overall, their findings led them to urge assessments of (investigator) error in similar future analyses. They surveyed relevant literature, noting that only 32%–35% of sampled studies conducted sensitivity analyses or model evaluation.

Bates et al. (2021) used the same specimens (and biomechanical models) as Broyde et al. (2021), but estimated PCSA (and volume) from muscle AA on the skull, using the dry skull method described above as well as a muscle‐specific method. They found large errors in estimated PCSA, usually underestimation, which had knock‐on effects on estimated bite forces and bone stresses. Each of the three rodent species' jaw muscle data were from one specimen and analysed by an unspecified investigator(s). These errors varied tremendously between muscles and species. The study inferred that (rodent) jaw muscle PCSAs might not be safely inferred from AA, and volumetric approaches such as by Broyde et al. (2021) may then be superior. They cautioned that such approaches using PCSA:AA relationships may lack ‘high fidelity’ for comparative or evolutionary reconstructions in general, and there may be a misleading assumption in the field that these relationships are straightforward.

In one of the few non‐mammalian examples, Sellers, Middleton, et al. (2017) measured *Alligator* jaw muscle AAs and estimated PCSAs using 3D frustral volumes, aimed at producing a method applicable to fossil archosaurs (the clade including Crocodylia, Aves and all descendants of their most recent common ancestor; e.g. Gauthier, 1986). Much like Thomason (1991) and subsequent studies, they found a good match between empirical and theoretical bite forces and their models, bolstering confidence in the PCSA:AA assumptions. They noted, however, that M. pterygoideus ventralis forces were underestimated by not accounting for aponeurotic (i.e. non‐skeletal) AAs; a reminder that attachment type (bony, tendinous, aponeurotic) is critical for PCSA:AA estimation. Their subsequent studies have expanded on these concepts and methods (Cost et al., 2022; Sellers et al., 2022). Bates and Falkingham (2012) (also see Gignac & Erickson, 2017) adopted a similar 3D modelling approach for estimating bite forces in a human and the archosaurs *Alligator* (juvenile and adult models) and *Tyrannosaurus*, estimating PCSAs from muscle volumes spanning approximate AAs; and again obtaining reasonable matches of empirical bite force data from humans and *Alligator*; although PCSA estimates tended to have 5%–12% error (22% maximum) investigated with sensitivity analyses. Similarly, Snively and Russell (2007) and Snively et al. (2013) reconstructed head and neck muscle dimensions in the theropods *Tyrannosaurus* and *Allosaurus*, but without testing the accuracy of those reconstructions in detail—although approximate data on PCSA:AA from some extant sauropsids were employed. Gröning et al. (2013) showed how having accurate, ideally subject‐specific data on muscle architecture was vital for obtaining good results from simulating bite forces in lizards. Most recently, Sakamoto (2022) used phylogenetic methods to predict jaw adductor muscle PCSA from skull width (based on a dataset from five extant bird species and extinct archosaurs), with good success.

If the correlations between PCSAs and AAs that have been found for some jaw muscles in some taxa also apply to other musculoskeletal systems such as limbs, and in different taxa, such correlations could provide powerful insights into not only basic form‐function relationships but also locomotor behaviour, adaptation, evolution and development. Yet to our knowledge these correlations have not yet been examined in detail for the limbs of tetrapods (but see Fahn‐Lai et al., 2020). Other studies have used alternative methods such as scaling limb muscle architecture from humans to fossil hominins (Wang et al., 2004) or from extant amniotes to fossil synapsids (Fahn‐Lai et al., 2020; see below). Thus an opportunity presents itself to address this question with new data and for a different clade.

Archosaurian reptiles represent a lineage with a remarkable disparity of their appendicular musculoskeletal system that has long fascinated scientists (e.g. Alexander, 1985; Charig, 1972; Cuff et al., 2019, 2022; Dilkes, 1999; Gatesy, 1990; Gatesy & Dial, 1996; Hutchinson, 2001; Hutchinson & Gatesy, 2000; Maidment & Barrett, 2011, 2012; Moore et al., 2022; Rhodes et al., 2021; Romer, 1923a, 1923b, 1923c; Walker, 1977; Wiseman et al., 2021). The ancestral condition for Archosauria was quadrupedal with enlarged pelvic bones and possibly a more erect (less sprawling posture), parasagittal gait (Charig, 1972; Gatesy, 1990; Sereno, 1991), powered by large caudofemoral tail muscles attaching to a fourth trochanter on the femur (Dollo, 1883; Gatesy, 1995; Persons & Currie, 2011; Romer, 1923a, 1923b, 1927). Indeed, some success in estimating the mass of the M. caudofemoralis longus in saurian reptiles and applying this method to extinct archosaurs has been obtained by prior studies (Allen et al., 2013; Bates et al., 2012; Hutchinson et al., 2011; Persons & Currie, 2011). Dinosaurs and their closest relatives eventually evolved bipedalism and greater cursorial limb structure and function along with an erect, digitigrade posture and stance and derived limb kinematics, before the K‐Pg mass extinction reduced the diversity of functions to that seen in birds today (e.g. Gatesy, 1990). There has been much interest in testing how extinct archosaurs moved and how major transitions in locomotion happened, including using biomechanical and musculoskeletal models (Alexander, 1985; Allen et al., 2021; Bishop, Falisse, et al., 2021; Charig, 1972; Cuff et al., 2019, 2022; Dilkes, 1999; Gatesy, 1990; Gatesy & Dial, 1996; Gregory & Camp, 1918; Hutchinson, 2004a, 2004b; Rhodes et al., 2021; Romer, 1923a, 1923b, 1923c; Wiseman et al., 2021). Much of this interest ultimately stems from the radical transformations of musculoskeletal morphology across archosaurian evolution (see references above), raising the questions about how form relates to function across such transformations.

Fahn‐Lai et al. (2020) measured PCSAs in lizard and mammal shoulder musculature, finding generally conservative architecture in amniotes that justified estimating the shoulder muscle PCSAs of the stem mammal *Massetognathus*, mainly using scaling equations. Similarly, Martin et al. (2019) found overall strong correlations between forelimb muscle PCSAs and bone shape (including indices of AAs) in the Quenda marsupial *Isoodon*. Importantly, Fahn‐Lai et al. (2020, their Table S5) showed that apomorphic synapsid muscles maintained somewhat conservative ratios of PCSAs to AAs, providing an alternative approach for PCSA estimation in fossil synapsids. Similarly, because archosaur hindlimb musculature changed dramatically across the crocodylian and avian stem lineages (references above), we expect that a similar approach of estimating PCSAs from AAs will generally be necessary (see also Bishop, Cuff, et al., 2021).

Fundamentally, all qualitative and quantitative inferences about how extinct archosaurs moved to depend upon some understanding of the magnitude of forces their limbs might have generated. Recent approaches have tried abstracting ‘antigravity’ and other muscles acting around joints to general masses, pennation angles and fascicle lengths scaled from extant taxa (e.g. Bates et al., 2010; Gatesy et al., 2009; Hutchinson, 2004a, 2004b; Hutchinson & Garcia, 2002; Sellers & Manning, 2007; Sellers et al., 2013) or even partitioning total muscle masses into individual muscle masses or other parameters (Bishop, Cuff, et al., 2021; Sellers, Pond, et al. 2017). Snively et al. (2019) estimated hip muscle areas in theropod dinosaurs for approximating moment‐generating capacity useful in turning, assuming that PCSA and AA were consistently related across muscles and taxa. In a more detailed study, Rhodes et al. (2021) digitised two two‐dimensional views of reconstructed muscle AAs in extant and extinct saurians, with a focus on maniraptoran theropods, roughly assuming that these AAs had some correlation with PCSA but acknowledging that this was a tentative abstraction. A key problem is that the underlying accuracy of these abstractions or partitions and their plausibility in light of reconstructed musculoskeletal anatomy remain to be tested. Most recently, however, Demuth et al. (2022) presented a new polygonal modelling approach for volumetric estimates from which PCSA could be derived, relying on skeletal dimensions to loft those volumes from and to. They found good agreement between their estimates and dissection‐based muscle mass measurements for crocodile hindlimb and gorilla shoulder muscles; and applied this method to the hindlimbs of the Triassic archosaurifom *Euparkeria capensis*. This approach (similar to the volumetric method of Herbst et al., 2022) is an attractive alternative, or complementary method; indeed, all methods could be complementary.

The aforementioned major transitions in archosaurian locomotor function and underlying musculoskeletal anatomy, and the interest in biomechanical models and simulations to address those big questions in archosaur evolution, provoke our study's main question: how well do archosaurian hindlimb muscle AAs correlate with their corresponding PCSAs? For over a century, to some degree, it has at least implicitly been assumed that there is a good correlation between AA and PCSA for archosaurian pelvic appendicular muscles (e.g. Bishop, Cuff, et al., 2021; Dilkes, 1999; Gatesy, 1990; Gregory & Camp, 1918; Moore et al., 2022; Rhodes et al., 2021; Romer, 1923a, 1923b, 1923c; Sellers, Pond, et al. 2017). To answer this question, we gathered a dataset of crocodylian and avian hindlimb muscle AA and PCSA data from dissections to identify how well each muscle's AA matched its PCSA in each specimen and taxon. We then applied these results to digitised AA estimates for a reconstruction of the Late Triassic theropod dinosaur *Coelophysis bauri* Cope 1887 to compare the PCSA estimates from our new methodology to those from a semi‐independent method (Bishop, Cuff, et al., 2021). This leads to our study's second question, which is how different methods for estimating muscle PCSA and mass compare in terms of advantages and disadvantages, which we discuss more broadly in light of our findings from our first question.

## 2 MATERIALS AND METHODS

### 2.1 Specimens

Our main specimens were five juvenile female Nile crocodiles (*Crocodylus niloticus*) and six adult Elegant‐crested tinamou birds (*Eudromia elegans*). We supplemented this main sample with additional single specimens each of an adult ostrich (*Struthio camelus*), emu (*Dromaius novaehollandiae*), wild turkey (*Meleagris gallopavo*) and domestic chicken (*Gallus gallus*). Table 1 lists subject information. Animals were obtained from prior studies (Bishop, Michel, et al., 2021; Cuff et al., 2019; Hutchinson, 2004a; Wiseman et al., 2021) in which they had been euthanised via humane methods or donated for our research from humane euthanasia at local farms (the four additional bird specimens; from Hutchinson, 2004a; Hutchinson et al., 2015). All specimens had been frozen at −20°C after death and maintained so until dissection, then thawed for 24+ h at 4°C until ice‐free but still cool to the touch. When not being dissected they were kept cool (refrigerated at 4°C) and moist (in a box wrapped in damp towels) to avoid desiccation.

**TABLE 1:** Subjects used in this study

Subject	Body mass (kg)
DDNC04	3.399
DDNC06	2.977
DDNC07	3.000
DDNC08	1.830
DDNC10	6.100
DDT01	0.525
DDT04	0.540
DDT05	0.700
DDT08	0.528
DDT12	0.620
DDT13	0.666
OO	65.3
OE	27.2
OT	3.70
ODC	4.02

Abbreviations: DDNC, Nile crocodiles; DDT, Elegant‐crested tinamous; ODC = domestic chicken; OE, emu; OO, ostrich; OT = wild turkey.

### 2.2 Dissections, digitisation and data processing

**FIGURE 1:**
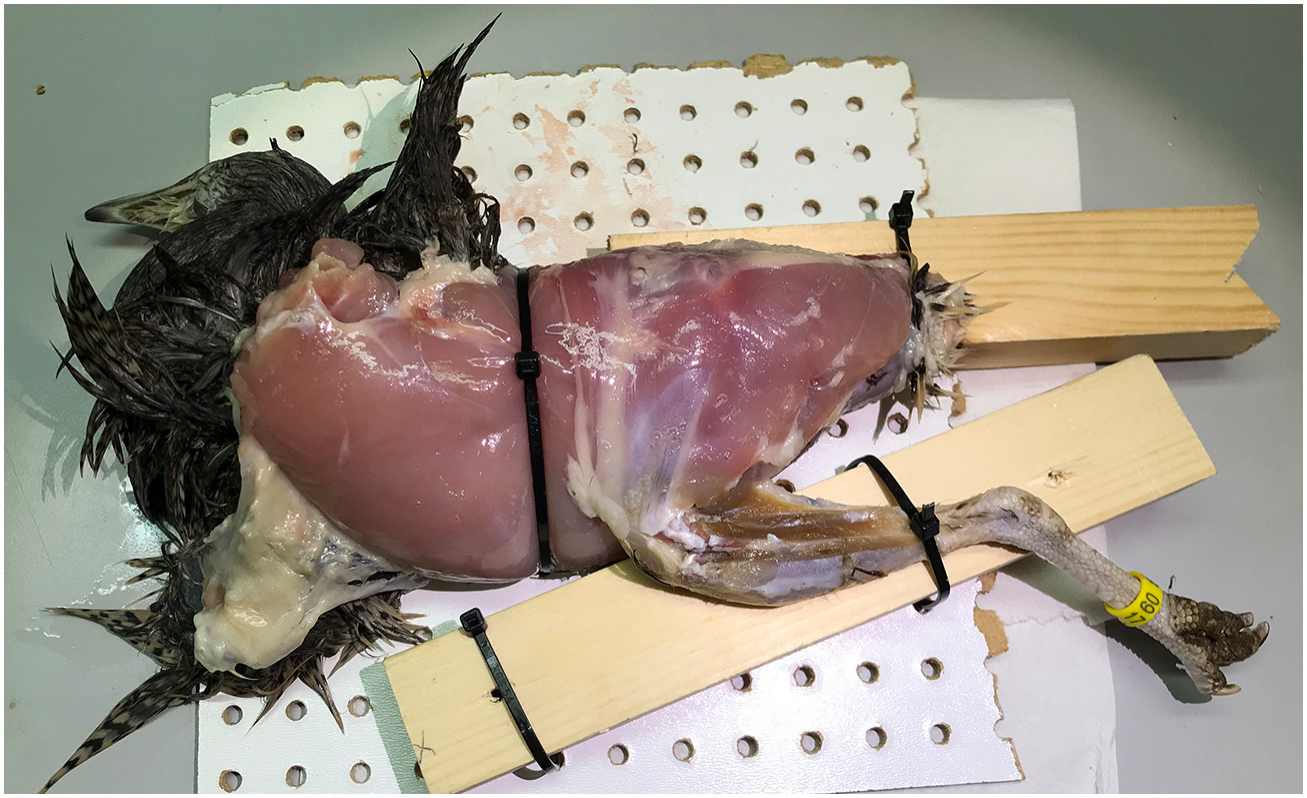
Tinamou fixed to corkboard with cable ties so that the lower limb was held stable relative to the body

We first dissected specimens to identify all major hindlimb muscle groups down to the ankles (here we did not study intrinsic foot muscles that originate on the tarsals/tarsometatarsus or distal to them). Our analyses here concentrate on muscle origins rather than insertions (with one exception noted later), as we deemed the latter to be too small (hence more measurement error‐prone), and because of their small size (especially in birds) perhaps too difficult to discern from other soft tissues, to obtain reliable data in most cases. There was also the pragmatic reason that digitising insertions would (as quite a few lower limb muscles have more than one insertion) more than double the digitising time, and thereby heighten the risk that soft tissue decay would reduce the quality of data. We expected some differences between more ‘direct’ (fleshy/muscular; often fibrous) vs. more ‘indirect’ (tendinous/aponeurotic; often fibrocartilaginous) attachments (Benjamin et al., 1995; Rothschild et al., 2015; Woo et al., 1988); as well as differences between proximal (more parallel‐fibred; less tendinous and less tapered, more fusiform in shape) and distal (more pennate, tendinous and triangular) muscles, which our statistical methods examined. We fixed specimens in a stable position using cable ties and corkboard clamped to the table surface, so that one appendicular segment at a time was held immobile (Figure 1). The individual muscles were separated away from neighbouring muscles so that the individual origins were accessible.

Digitisation of the perimeters for individual muscle origins proceeded using a Microscribe X digitiser (±0.01 mm) for our main specimens (in Rhinoceros software; McNeel Europe, Barcelona, Spain) or a custom‐built digitiser (±1 mm) (as in Hutchinson et al., 2015) to begin collection of the AAs of each muscle, emphasizing the bony origins alone (i.e. avoiding digitisation of any parts of the origin from soft tissue such as fascia, tendon or surrounding muscle/skin). Where the attachment was over a large or complex area (e.g. wrapping bones), areas within the AA perimeter were also digitised to better map the 3D geometry of the AA. We removed muscles in our sample that had ‘point’ attachments; i.e. deemed too small to digitise in an accurate, meaningful way beyond ~1 point. For our main sample muscles tended to be 10–60 points of total digitisation depending on the size and complexity of the origin (e.g. wrapping around limb bones creating curvatures); for the additional avian sample, the number of points varied but was generally fewer, approximately 13–30. Digitised data were exported as (x, y, z) point cloud text files for further analysis. The four birds other than the tinamous used different digitiser hardware and slightly different methodology, as detailed by Hutchinson et al. (2015).

**FIGURE 2:**
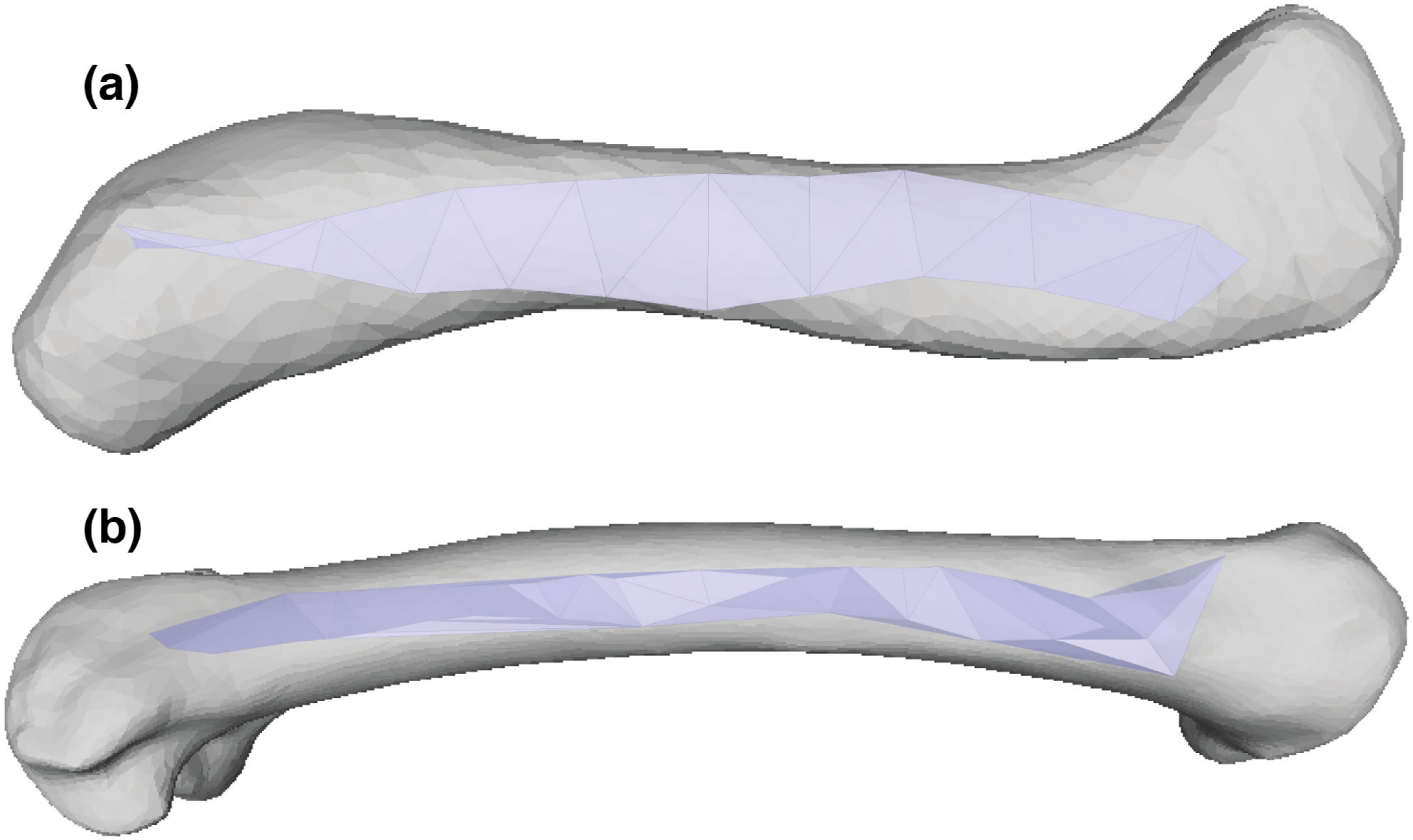
Examples of final triangulated OBJ meshes, for homologous (a) *Crocodylus* M. femorotibialis externus and (b) *Eudromia* M. femorotibialis lateralis muscle origins (lateral view; proximal on right side); in Meshlab. Superimposed onto left femur (from Bishop, Michel, et al., 2021; Wiseman et al., 2021) in approximate relative orientation. Not to scale.

**FIGURE 3:**
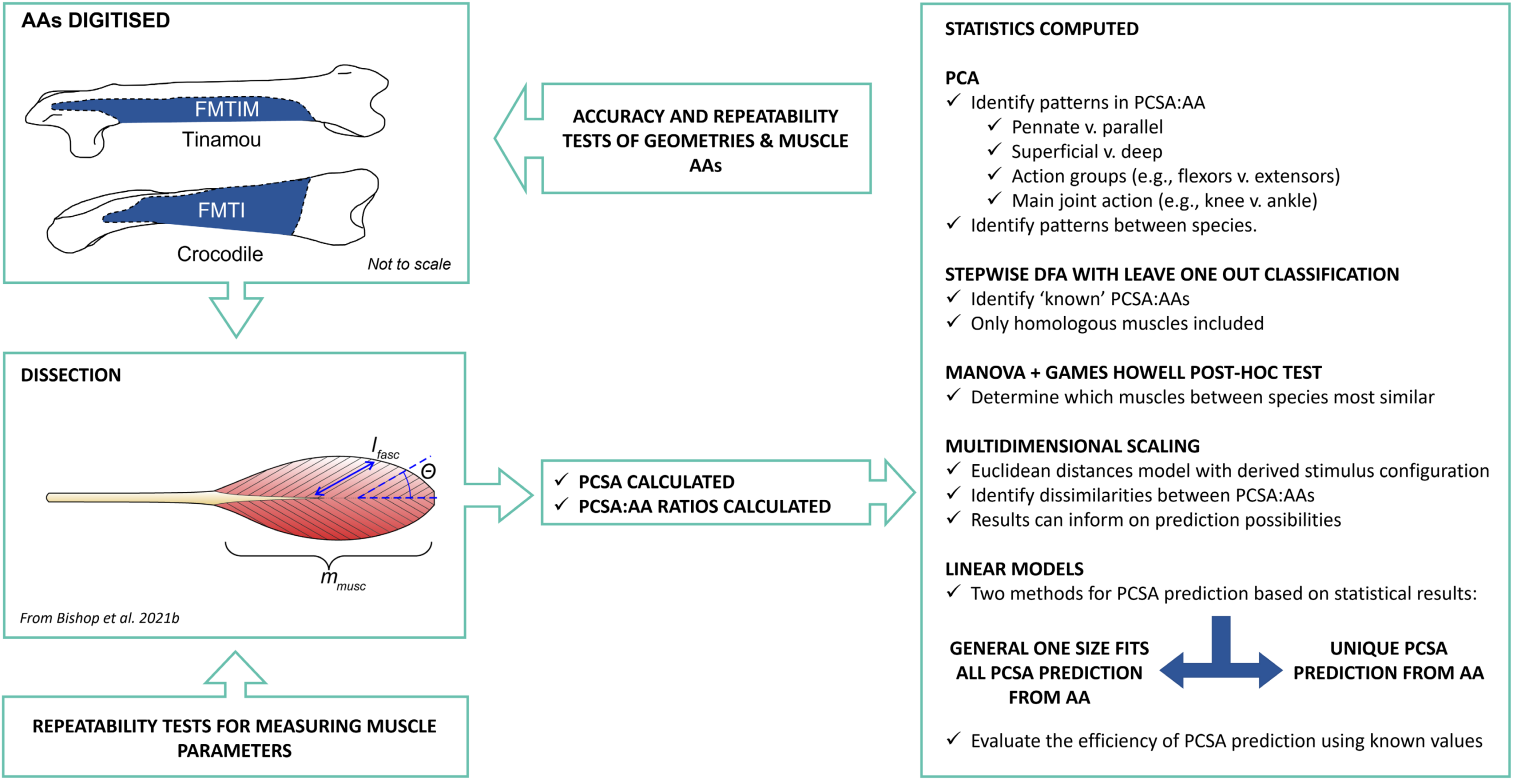
Flowchart of data collection and statistical protocols followed in this study, in which muscular attachment sites were digitised and then dissected (symbols as in Equation 1). The PCSA of each muscle was calculated, after which several statistical analyses were computed to (1) determine if a ‘one size fits all’ approach could be used to predict PCSA from AA of muscles, or if some muscle groups instead have a unique relationship between PCSA and AA requiring muscle‐specific approaches; and (2) establish how to predict PCSA from AA.

All AA data were analysed similarly. A custom script (shared at https://figshare.com/projects/Anatomically_Grounded_Estimation_of_Hindlimb_Muscle_Sizes_in_Archosauria/144135) for MATLAB (The MathWorks, Inc.) was used to produce a triangulated mesh from the point cloud data using Delaunay triangulation for the semi‐planar AAs (i.e. most muscle origins studied). The plane of triangulation (i.e. x–y, y–z or z–x) was subjectively chosen as that which produced the smoothest mesh with the fewest spurious triangles. Even then, as Delaunay triangulation produces a convex shape, this can result in extraneous triangles around the periphery of a non‐convex muscle outline; these extraneous triangles were manually removed in Meshlab (http://www.meshlab.net/). For a few AAs that were markedly curved in space, such as those of the femorotibialis (which wraps around much of the femoral diaphysis), an alpha shape was used to generate a tight‐fitting triangular mesh from the point cloud instead; again, any extraneous triangles generated in this step were identified and removed in Meshlab. The final triangulated surfaces (e.g. Figure 2) were saved as OBJ format files and surface area was computed in Meshlab. We compiled all AA data into a spreadsheet in Excel software (Microsoft Office; Microsoft Inc.) for analyses. Figure 3 shows this digital workflow.

To examine our digitising protocol's precision and accuracy, we conducted two tests. First, we created geometry of known areas in Rhinoceros software: a circle, an eight‐pointed star and an arbitrary irregular shape (even more haphazardly shaped than a normal muscle origin should be), connected by curves/lines (Figure S1). These respectively were chosen as a ‘best case’ or gold standard, where the circle should be easily digitised and its area correctly calculated; then a ‘worse case’, where the star's points might cause the area to be overestimated because polygons would be added to connect the star's tips; then an ‘unknown case’ where the irregular shape had unpredictable (but we expected likely to be overestimated) errors due to its complexity (more like a real muscle's). We computed and recorded these areas in Rhinoceros. Next, we printed this geometry onto A4 paper at 5× scale and digitised them with ~30 points via the same Microscribe hardware, five times each (same procedure as for muscles above). This was used to quantify the broad range of errors that different geometries might cause, even under fairly ideal conditions of perfectly 2D shapes and very clear boundaries (unlike muscle AAs).

Second, we digitised three right hindlimb muscle origins of a subadult specimen (2.17 kg body mass) of *Caiman crocodilus* (cadaver donated from same origin; La Ferme Aux Crocodiles, Pierrelatte, France): Mm. femorotibialis internus (FMTI), iliofemoralis (IF) and iliotibialis 2 (IT2). These were chosen for their shapes and sizes, representing some of the diversity of AAs studied: the FMTI being large, curved and elongated on the femoral shaft; the IF being smaller and rounded in shape on the lateral ilium; and the IT2 being thin and elongate on the dorsolateral ilium. These were also digitised five times each, ~30/20/15 points for FMTI/IF/IT2. This approach was used to quantify the repeatability of measuring crocodylian AA.

For both datasets of geometry and muscle areas, we ran the same Delaunay triangulation procedure in MATLAB as described above. However, we did not edit the OBJ meshes produced for the ‘known’ geometry to remove stray polygons, because we judged that this would only bias the analysis's accuracy (i.e. we would know exactly the geometry desired). Again, we calculated the resulting areas in Meshlab.

Subsequently, we made PCSA calculations following standard muscle architecture techniques (e.g. Cuff et al., 2016) and equation 1:

(1) $$\text{PCSA}=\frac{m_{\text{musc}}\cos\;\Theta }{l_{\text{fasc}}\;d}\;$$

where *m*_musc_ = muscle mass (g), Θ = pennation angle relative to the muscle's line of action, *l*_fasc_ = fascicle length, and *d* = muscle density (=1060 kg m^−3^; Mendez & Keys, 1960). We cut away tendons and other non‐muscular tissue, then weighed muscle masses on an electronic balance (g), cutting the muscle open as necessary to reveal muscle fascicles, and took 3–15 measurements/muscle for Θ and *l*_fasc_. These PCSA data were finally added to the above spreadsheet.

### 2.3 Statistical Analyses

All statistical analyses were computed in IBM SPSS 28.0.0. We focused on our main sample of Nile crocodiles and tinamous as these data were all collected with the same tools and methods and by the same individual (for crocodiles/tinamous respectively), having agreed on a protocol in advance and tested it together to ensure repeatability of the basic methods. The additional avian sample was gathered for purposes other than this study and the resolution of data was expected to be lower due to less rigorous protocol in measurements, so we only combined these two datasets tentatively in later analyses, in an exploratory fashion to determine if all avian data were similar or dissimilar, allowing us to identify if a ‘one size fits all’ approach for estimating PCSA from AA in Aves was possible. Our statistical analyses focused on the ~31 major muscle groups identified by homologies in Table 2.

**TABLE 2:** Major hindlimb muscles of Archosauria, with names for homologous muscles in Crocodylia and Aves, and corresponding abbreviations used here. Archosaur hindlimb homologies adopted follow Hutchinson (2002) and references therein, and Hattori and Tsuihiji (2021) for distal limb muscles (e.g. TA/EDL, FDL/FHL)

Muscles (Crocodylia)	Abbreviation	Muscles (Aves)	Abbreviation
*M. iliotibialis 1*	IT1	*M. iliotibialis cranialis*	IC
*M. iliotibialis 2*	IT2	*M. iliotibialis lateralis pars preacetabularis*	AIL
*M. iliotibialis 3*	IT3	*M. iliotibialis lateralis pars postacetabularis*	PIL
*M. femorotibialis externus*	FMTE	*M. femorotibialis lateralis*	FMTL
**[FMTI split in/before Aves]**	—	*M. femorotibialis intermedius*	**FMTIM**
*M. femorotibialis internus*	FMTI	*M. femorotibialis medialis*	**FMTM**
*M. ambiens 1*	AMB1	*M. ambiens*	AMB
*M. ambiens 2*	**AMB2**	**Absent (except analogue in ostrich); presumed autapomorphy of Crocodylia**	**(AMBd)**
*M. iliofemoralis*	IF	*M. iliotrochantericus caudalis*	**ITC**
**[IF split in Dinosauromorpha]**	—	*M. iliofemoralis externus*	**IFE**
*M. puboischiofemoralis internus 1*	PIFI1	*M. iliofemoralis internus*	IFI
*M. puboischiofemoralis internus 2*	PIFI2	*M. iliotrochantericus cranialis*	**ITCR**
**[PIFI2 split in/before Aves]**	—	*M. iliotrochantericus medius*	**ITM**
*M. iliofibularis*	ILFB	*M. iliofibularis*	ILFB
*M. flexor tibialis internus 1*	**FTI1**	**Absent; presumed plesiomorphy for Archosauria**	—
*M. flexor tibialis internus 2*	**FTI2**	**Absent; presumed plesiomorphy for Archosauria**	—
*M. flexor tibialis internus 3*	FTI3	*M. flexor cruris medialis*	FCM
*M. flexor tibialis internus 4*	**FTI4** ^a^	**Absent; possible autapomorphy of Crocodylia**	—
*M. flexor tibialis externus*	FTE	*M. flexor cruris lateralis pars pelvica*	FCLP
**Absent; presumed autapomorphy of (or more inclusive than) Aves**	—	*M. flexor cruris lateralis pars accessoria* ^a^	FCLA^a^
*M. adductor femoris 1*	ADD1	*M. puboischiofemoralis medialis*	PIFM
*M. adductor femoris 2*	ADD2	*M. puboischiofemoralis lateralis*	PIFL
*M. puboischiofemoralis externus 1*	PIFE1	*M. obturatorius lateralis* ^a^	OL^a^
*M. puboischiofemoralis externus 2*	PIFE2	*M. obturatorius medialis*	OM
*M. puboischiofemoralis externus 3*	**PIFE3**	**Absent; presumed plesiomorphy for Archosauria**	—
*M. ischiotrochantericus*	ISTR	*M. ischiofemoralis*	ISF
*M. caudofemoralis brevis*	CFB	*M. caudofemoralis pars pelvica*	CFP
*M. caudofemoralis longus*	CFL	*M. caudofemoralis pars caudalis*	CFC
*M. gastrocnemius internus*	GI	*M. gastrocnemius (pars) medialis*	**GM**
**[GM split in/before Aves]**	—	*M. gastrocnemius (pars) intermedius*	**GIM**
*M. gastrocnemius externus*	GE	*M. gastrocnemius (pars) lateralis*	GL
*M. extensor digitorum longus*	EDL	**Split into *M. tibialis cranialis* ‐ *caput femorale* and *caput tibiale* in/before Aves [Hattori & Tsuihiji** **2021** **]**	**TC(f + t)**
*M. extensor hallucis longus* ^a^	EHL^a^	*M. extensor hallucis longus* ^a^	EHL^a^
*M. extensor digitorum brevis* ^a^	EDB^a^	**Ancestral TA and EDB fused in/before Aves to form *M. extensor digitorum longus* [Hattori & Tsuihiji** **2021** **]**	**EDL**
*M. tibialis anterior*	TA		
*M. flexor digitorum longus*	FDL	*M. flexor hallucis longus* (+ *flexor perforans* muscles)	FHL
*M. flexor hallucis longus*	FHL	*M. flexor digitorum longus*	FDL
*M. fibularis longus*	FL	*M. fibularis longus*	FL
*M. fibularis brevis*	FB	*M. fibularis brevis*	FB
*M. interosseous cruris*	IOC	*M. popliteus* ^a^	POP^a^
*M. pronator profundus*	PP	**Absent; presumed plesiomorphy for Archosauria**	—

*Note*: For emphasis, muscles in bold font either are split into multiple heads in Crocodylia/Aves or are absent in one of them.

^a^ Muscle not studied here, but included in this table for comparative context (remaining muscles were studied here).

We were curious how consistently we measured fascicle lengths within our sampled muscles. Charles et al. (2022) showed that small sample sizes of fascicle lengths can be poorly representative of architecture, having knock‐on effects for analysis of muscle function; our test partly addresses this concern. Different fascicle lengths throughout each muscle were measured at least three times on the same day by the same individual, following standard approaches (e.g. Martin et al., 2020). To test the consistency of measuring fascicle lengths across a muscle's volume, an rANOVA was computed on all fascicle length measurements in both crocodiles and tinamous. The accuracy, precision and repeatability tests described above for ‘known’ geometry and three *Caiman* muscle AAs were checked purely via descriptive statistics.

Our first main question focused on how similar the PCSA:AA ratios were within a species. PCSA:AA variation in crocodile‐only musculature (*n* = 5 crocodiles; five observations per muscle PCSA:AA) was assessed using a principal component analysis (PCA) to determine patterns of similarity/dissimilarity in PCSA:AA. Four different categorical variables were introduced which were treated as independent observations to identify which factor(s) explained most of the variation: (1) muscles were qualitatively assigned a categorical variable based on their ‘main’ function (i.e. hip flexors vs. hip extensors); (2) muscles were categorically grouped according to their classification as parallel or pennate; (3) superficial vs. deep muscles were grouped together; and (4) muscles were grouped according to the primary joint around which they acted (i.e. hip, knee, ankle and toe muscles).

Variation between Crocodylia and Aves was first assessed using a between‐groups PCA. This methodology allows the number of variables to be higher than the number of observations (Mitteroecker & Bookstein, 2011), which was particularly relevant for comparative analyses of the crocodile versus the Aves groups, which had missing dissection data for some muscles, in addition to unequal group sizes (e.g. five PCSA:AA ratios per muscle for the crocodiles, versus the one PCSA:AA ratio for the turkey). The purpose of this statistical test was not to draw any statistical comparisons between individual species, but rather to determine if Crocodylia and Aves data were comparably similar or dissimilar, thus permitting the following analyses. Two tinamou specimens were excluded for this between‐groups PCA test because of reduced sample sizes of dissection data relative to the other specimens: DDT12 (*n* = 3 muscles) and DDT13 (*n* = 9 muscles).

Next, we tested if we could successfully predict ‘known’ PCSAs from AAs in our crocodile and Aves dataset. We used a stepwise Discriminant Function Analysis (DFA) with a leave‐one‐out classification to control for uneven sample sizes (Huberty, 1994; Lance et al., 2000). We could only include muscles which were strictly homologous, having one equivalent head in both groups, for crocodiles and Aves (Table 2). We also excluded muscles which had missing dissection data (i.e. the FCLP/FTE was only dissected in one tinamou specimen, with additional missing measured parameters in other Aves specimens for the same muscle). Therefore, we focussed only on 15 muscles' PCSA:AAs in crocodiles, tinamous and other avian specimens (turkey, emu, ostrich and chicken) to determine if those specific homologous muscles were distinguishable between each of the groups.

A multivariate analysis of variance (MANOVA) with a Games–Howell Post‐Hoc test was computed on these 15 homologous single‐headed muscles, using species assignment as a fixed effect. This MANOVA determined the statistical significance of variation in PCSA:AA amongst the different species (crocodiles, tinamous and the other measured Aves species). Because the MANOVA method was limited to only 15 equivalent single‐headed muscles in Crocodylia and Aves, we processed all muscle data for the crocodiles, tinamous and other avian species through a multidimensional scaling analysis (MDS) using Euclidean distances model with a derived stimulus configuration, following the Kruskal algorithm (Kruskal, 1964). MDS is a visual representation of dissimilarities between sets of objects using the mean of the ratio for all species. This method permitted us to include all ~31 measured muscles.

Furthermore, we included the crocodylian M. caudofemoralis longus (CFL). We did not have PCSA:AA ratio data for this muscle's origin AA for multiple reasons: this was well outside the range of the digitiser arm; the CFL's substantial origin from surrounding soft tissue would complicate estimations of AA; and prior studies discussed in the Introduction (and below) already presented methods for reliably reconstructing the CFL origin. However, we did have the CFL's PCSA and obtained AA data for its large insertion on the fourth trochanter of the femur. We included this in order to test if the CFL's PCSA, in this case, might be estimated from the AA of insertion; not origin. If so, this would complement alternative digital volumetric modelling methods to estimate mass or PCSA of the CFL (Bates et al., 2012; Hutchinson et al., 2011; Persons & Currie, 2011) and help circumvent the problem that CFL's PCSA might be greatly underestimated using some methods (see Bishop, Cuff, et al., 2021).

After identifying patterns of similarities and dissimilarities in PCSA:AA between species, we produced different sets of prediction equations to estimate PCSA from AAs using a series of linear regressions. Data were logged to normalise them and reduce skewness. These equations were (1) a ‘one size fits all’ equation for identifiably similar PCSA:AAs and (2) unique (muscle‐specific) regression equations for individual muscles which were identifiably outliers in the dataset:

(2) PCSA=m*AA+c

where *m* = slope of the linear regression and *c* = y‐intercept. A flowchart detailing the statistical protocol in this paper is in Figure 3.

Finally, to test how well we could predict PCSA from the AA of muscles, we computed the percentage difference between each predicted PCSA value and the measured dissected PCSA value, and then reported the likelihood of how many predicted muscles fell within a specified percentage (e.g. how many predicted PCSAs were predicted within <5% of their measured value versus how many were > 40% greater or smaller than their measured values). This approach permitted us to establish the accuracy of muscle PCSA prediction from AA, whilst also establishing the efficacy of the ‘one size fits all’ approach (i.e. if many muscles' PCSAs were predicted to be >50% from their original value, then we would conclude that this estimation was inaccurate, and that all muscles should use a unique approach).

### 2.4 *Coelophysis bauri* Case Study

Here we used the 3D computer model of Cleveland Museum of Natural History exhibit specimen number 10971 (see Allen et al., 2013; Bishop, Cuff, et al., 2021; Bishop, Falisse, et al., 2021), based on a moderate‐resolution laser scan of a mounted, composite exhibit skeleton (three adult individuals). We imported the skeleton of this model as. OBJ files into Rhinoceros software and used the *PolylineOnMesh* tool to digitally draw the perimeter of a given muscle's attachment directly onto the bone meshes; the polyline thus created was used to isolate the AA as its own separate mesh entity using the *SplitMeshWithCurve* tool. We attempted to remain as close as possible to the phylogenetically informed (but two‐dimensional) ‘muscle map’ reconstruction and musculoskeletal model of Bishop, Cuff, et al. (2021, their figure 5) using the approximate centroids of muscle attachments from that muscle map reconstruction. We also used the general 3D topography of the bones and relative topology of the muscle attachments (in extant archosaurs, and in the muscle map and the reconstruction itself) to guide the reconstruction (e.g. where muscles were with respect to the hip joint, fossae, trochanters, crests, condyles and other clear landmarks). However, we acknowledge that some of the areas in that muscle map (particularly for more distal parts of the limb) are less well constrained, as no direct osteological correlates of muscle attachment are preserved. We recorded the AA of each attachment mesh (measured via standard area function) from Rhinoceros into our spreadsheet for comparison to extant archosaurian data.

We also compared our PCSA estimates with the results of Bishop, Cuff, et al. (2021), who used normalised muscle architecture data (muscle masses, and then fibre lengths estimated from musculotendon length changes; Sellers et al., 2013, 2017) from extant archosaurs to estimate PCSAs (their ‘variant 3’ musculoskeletal model). As in the latter study, the maximal isometric force (*F*_max_) of muscles was estimated as (PCSA * 0.3 N mm^−2^) (Medler, 2002; Michel et al., 2020). Furthermore, we predicted the total single‐hindlimb muscle mass and PCSA from body mass (of *Coelophysis bauri*) using the phylogenetically generalised least squares regressions using the dataset of Bishop, Wright, et al. (2021) in R Studio (https://www.r‐project.org/). Those data were from humans and five avian species; see the study for more information. We compared these two predictions with variant 3's predictions, to test how well this method agreed with scaling data; as our dataset involved animals smaller than *Coelophysis bauri*.

## 3 RESULTS

### 3.1 General patterns for PCSA:AA ratios

Naively (or as a null assumption), one might expect PCSA:AA ratios close to 1. We found wide variation in the ratio of PCSA:AA across muscles and our two main study taxa, approximately varying one hundredfold from 0.10 to >10 (Table S1). We regressed PCSA against AA for all muscles/specimens (e.g. Equation 2). Our results demonstrated an overall poor correlation (Figure S2A; *R* ^2^ = 0.015) if all data were included. If outliers were removed, the correlation between the variables was improved, but remained very weakly associated (Figure S2B; *R* ^2^ = 0.0789). This was our first indication that a simple ‘one size fits all’ approach would not be possible for all muscles, but instead might be appropriate for a sub‐sample of our muscles such as those which clustered together in the regression. We computed additional statistical analyses to ascertain which muscles might share a consistent relationship between PCSA and AA and which muscles did not (see further below). These analyses eliminated further outliers from the dataset, producing a sub‐sample of hindlimb musculature which fit into a ‘one size fits all’ equation, in which PCSA and AA were positively correlated (Figure S2C; *R* ^2^ = 0.4872).

More indirect, ‘fleshy’ attachments (e.g. the three M. iliotibialis heads of birds; FMTE of Crocodylia; ILFB and PIFM or ADD2) tended to have PCSA:AA ratios closer to ~1, and more so in *Eudromia* than *Crocodylus* in general (~10 vs. 6 muscles with adequate data had ratios within 0.5–1.5). In contrast, muscles with small tendinous or aponeurotic origins often had greater or smaller PCSA:AA ratios (e.g. AMB1 ~13 and FTI3 ~10 in *Crocodylus*; FL ~4.3 in *Eudromia*; GE/GL >3–8 in both archosaurs).

Across our main sample, the grand mean PCSA:AA ratio was 3.17 for *Crocodylus*, 0.931 for *Eudromia* and 6.55 for ‘other Aves’; 3.07 overall; but with wide variation (standard deviations comparable to the means), so these general patterns are not emphasized here. Comparing proximal to distal hindlimb muscles in crocodiles vs. tinamous, we broadly found that birds were more disparate (proximal vs. distal: *Crocodylus*: 2.97 vs. 3.63; *Eudromia*: 0.591 vs. 1.84), having on average greater PCSA for a given AA value distally vs. proximally. Only 4/31 muscles (FMTI/(FMTIM+FMTM), ILFB, PIFI2/(ITCR+ITM), GI/GM) had PCSA:AA ratios for *Crocodylus* vs. *Eudromia* that were within the bounds of 0.5–1.5; others were more disparate.

However, these were simply casual observations of apparent trends in our averaged data. The following statistical tests explored the patterns in far more depth, allowing us to ultimately test how accurately we could predict PCSA from AA in our sample, and how the resulting approach compares with an alternative method (Bishop, Cuff, et al., 2021) for estimating muscle maximal force in the dinosaur *Coelophysis*.

### 3.2 How accurately was ‘known’ geometry digitised and how repeatable were muscle AAs?

Results of our accuracy/reliability tests are in Table S2. The circle, star and irregular shapes were digitised with ~4%, 229% and 218% error (overestimates). In contrast, standard deviations (SDs) were greatest for the circle (SD 0.77% of the mean) and least for the star (SD 0.41% of the mean), but the low SDs for all shapes indicated that small variances did not reflect overestimation error. The muscle origin AAs had considerable variation: SDs ranged from 13% (FMTI) to 36% (IT2) of the mean AAs.

### 3.3 How consistent were muscle fascicle lengths within muscles?

Using one‐way repeated measures ANOVAs, we found that there was no significant variability in our independent measurements of fascicle length for all muscles in each of the crocodile and tinamou samples (*F* ≤ 3.95; *p* ≥ 0.05 for all specimens). The maximum variability for all muscles from each specimen is also reported, establishing that the maximum standard errors of all fascicle lengths in crocodiles and tinamous were less than 2% or 6% respectively (Table 3).

**TABLE 3:** Consistency of measuring fascicle length in Nile crocodiles (DDNC) and Elegant‐crested tinamous (DDT)

		Variability		Repeated measures ANOVA					
Species	Specimen ID	Maximum variance (mm)	SE (%)	SS	df	MS	*F*	*p*	*F* Crit
Nile crocodile	DDNC04	6.70	1.50	643.571	3	321.785	1.153	0.322	3.132
	DDNC06	8.88	1.72	686.051	3	228.684	1.071	0.365	2.691
	DDNC07	4.70	1.09	435.225	4	108.806	1.013	0.403	2.435
	DDNC08	9.80	1.81	15.590	3	5.197	1.941	0.128	2.694
	DDNC10	5.51	1.36	24.816	3	12.408	0.166	0.847	3.132
Tinamou	DDT01	4.94	2.70	8.883	3	2.961	0.395	0.757	2.798
	DDT04^a^	3.18	4.93	86.832	3	28.944	0.839	0.477	2.727
	DDT05	5.78	4.72	39.165	2	19.583	1.070	0.355	3.295
	DDT08	6.80	4.39	38.382	3	12.794	0.674	0.571	2.737
	DDT12	3.34	2.22	53.743	3	17.914	2.948	0.120	4.757
	DDT13	7.44	6.08	21.115	3	7.038	0.586	0.628	2.827

*Note*: Maximum variance is for repeated measures for all muscles from that specimen.

Abbreviations: dF, degrees of freedom; MS, mean of squares; SE, standard error; SS, sum of squares.

^a^ The PIL muscle for DDT04 was removed because the muscle was unreliably measured (SE: 7.8%; muscle variability: 14.3 mm).

Because all muscles in general were established to be reliably measured, the following assessments were possible. We sub‐divided our results into the following sections: (1) Nile crocodile‐only analysis for which we had more dissected musculature data available and (2) comparative Crocodylia and Aves analysis to test if PCSA:AA was consistent across extant archosaurs studied.

### 3.4 How similar are the PCSA:AA ratios within Nile crocodiles?

**FIGURE 4:**
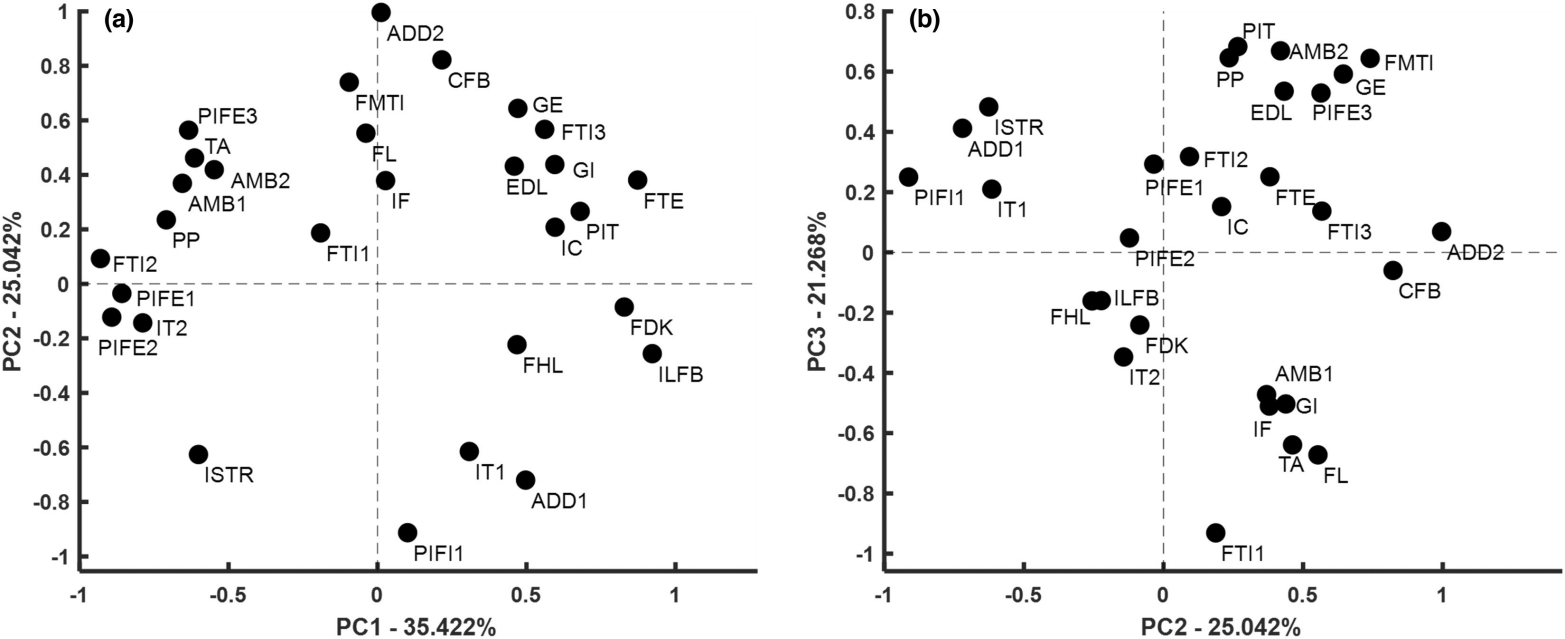
PCA graph of the Nile crocodile (*n* = 5) muscles (*n* = 29) along PC1, PC2 (together in a) and PC3 (vs. PC2 in b). Muscles were not grouped according to any categorical variable in this analysis as no patterning of data was identified, and thus variability was not associated with muscle location, fibre classification or muscle grouping.

The PCA for the crocodile‐only data (*n* = 5 specimens) produced a scattered mixture of positive and negative PC scores along three principal PC loadings (81.7% of variance), with poor separation of samples. Four unique PCSA:AAs were somewhat distinguishable along PC2 (explaining 25.0% of variance) and along PC3 (explaining 21.3% of the variance): the ISTR, IT1, ADD1 and PIFI1 PCSA:AA values. All of these outlying muscles would act around the hip and all are essentially parallel‐fibred muscles. The overlap in PC scores for all other muscles indicated that their PCSA:AA ratios were similarly proportionate; hence PCSA:AA was comparably similar in all 29 of the crocodylian muscles we studied (Figure 4).

When categorical variables were introduced to the PCA for the *Crocodylus niloticus* muscles, no patterns were identifiable. This indicated that the PCSA:AA ratios were not consistent between (1) primary muscle function, (2) classification as parallel vs. pennate, (3) superficial vs. deep muscle, (4) the primary joint around which the muscles acted (e.g. hip muscles) and (5) proximal vs. distal muscles (Figure 4).

### 3.5 PCA of similarity between PCSA:AA ratios of Crocodylia and Aves

**FIGURE 5:**
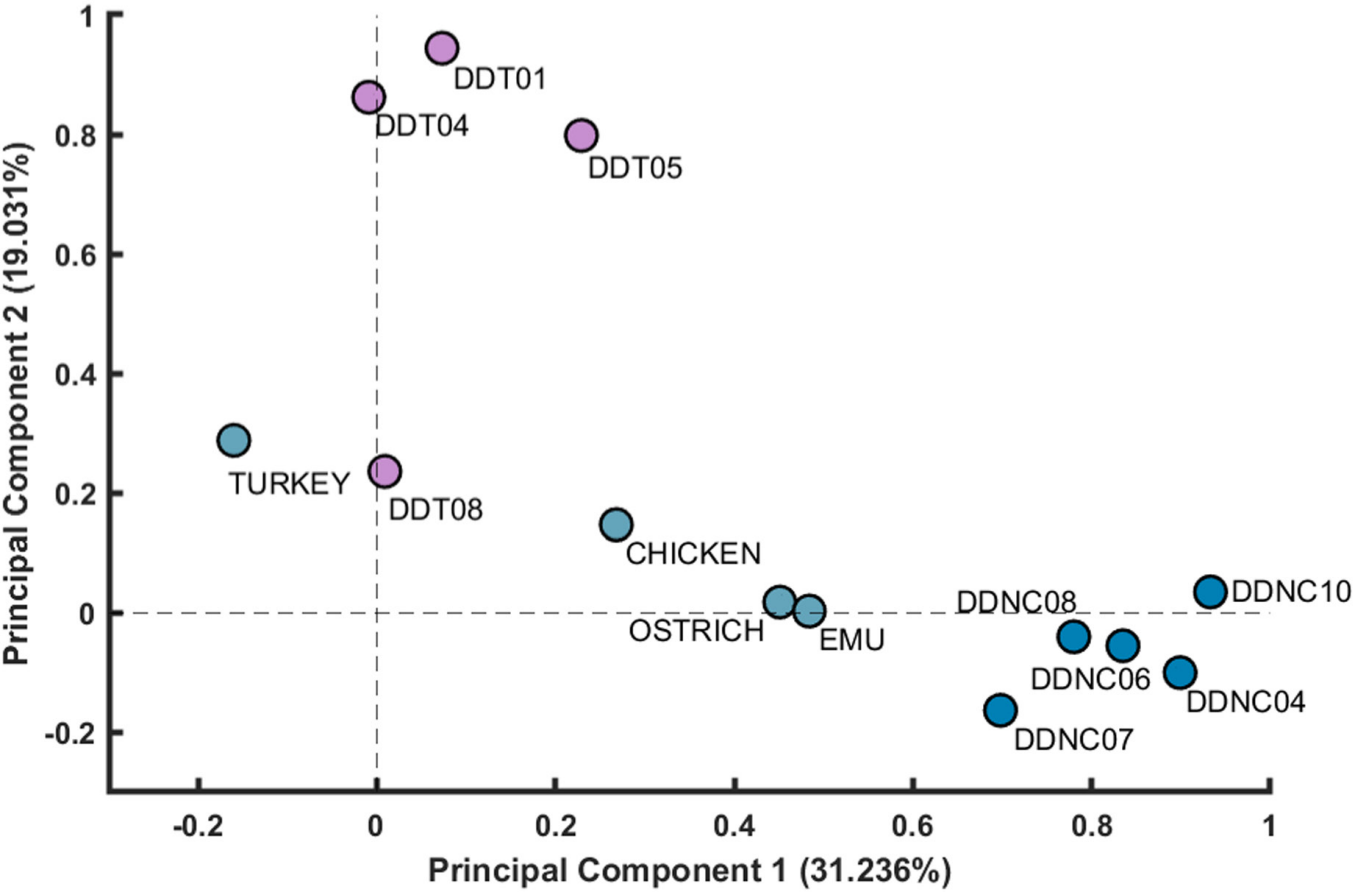
PCA plot of all grouped muscles within each species (birds and crocodiles). Categorical variables are used here to visualise different species, grouped according to the crocodiles, tinamous and other Aves. Crocodile data typically group well along both PC1 and PC2, whilst the tinamou and other Aves data are more variably distributed with poor grouping identified along both axes, indicating that Aves data are not subject‐ and/or species‐specific. Somewhat close similarity of the Aves data with the crocodiles along PC2 implies that all archosaur data might fit into a ‘one size fits all’ bracket, but a generally low sample size precludes such a generalised statement.

When a PCA for all Crocodylia and Aves was computed, separation along PC1 (31.3%) was characterised by separation of the crocodile specimens (positive scores) from other Aves specimens (mixture of positive and negative scores). There were further groupings evident in PC1: the chicken, ostrich and emu formed one group (positive scores), and all tinamou and the turkey formed another group (mixture of positive and negative scores). Separation along PC2 (19.0%) was characterised by separation of DDT01, DDT04 and DDT05 (positive scores) with all other specimens (mixture of positive and negative scores). Tentatively, we assumed that the separation of this group had different PCSA:AA ratios than all other specimens, although with an overall small sample size (*n* = 15 total individuals) we did not find this cause enough to exclude the data from those specimens (*n* = 3). The separation of groups along PC1 implies different PCSA:AA ratios (i.e. the crocodile ratios were distinct from the Aves). We identified inter‐ and intra‐specific variability in PCSA:AAs among extant archosaurs, as determined along PC2 and supported by the PCA reported for the crocodile‐only data (Figure 4). The overlap of data along PC2 in conjunction with a small sample size (*n* = 15) led us to conclude that separating archosaur data between Aves and crocodile groupings was not justified (Figure 5).

**FIGURE 6:**
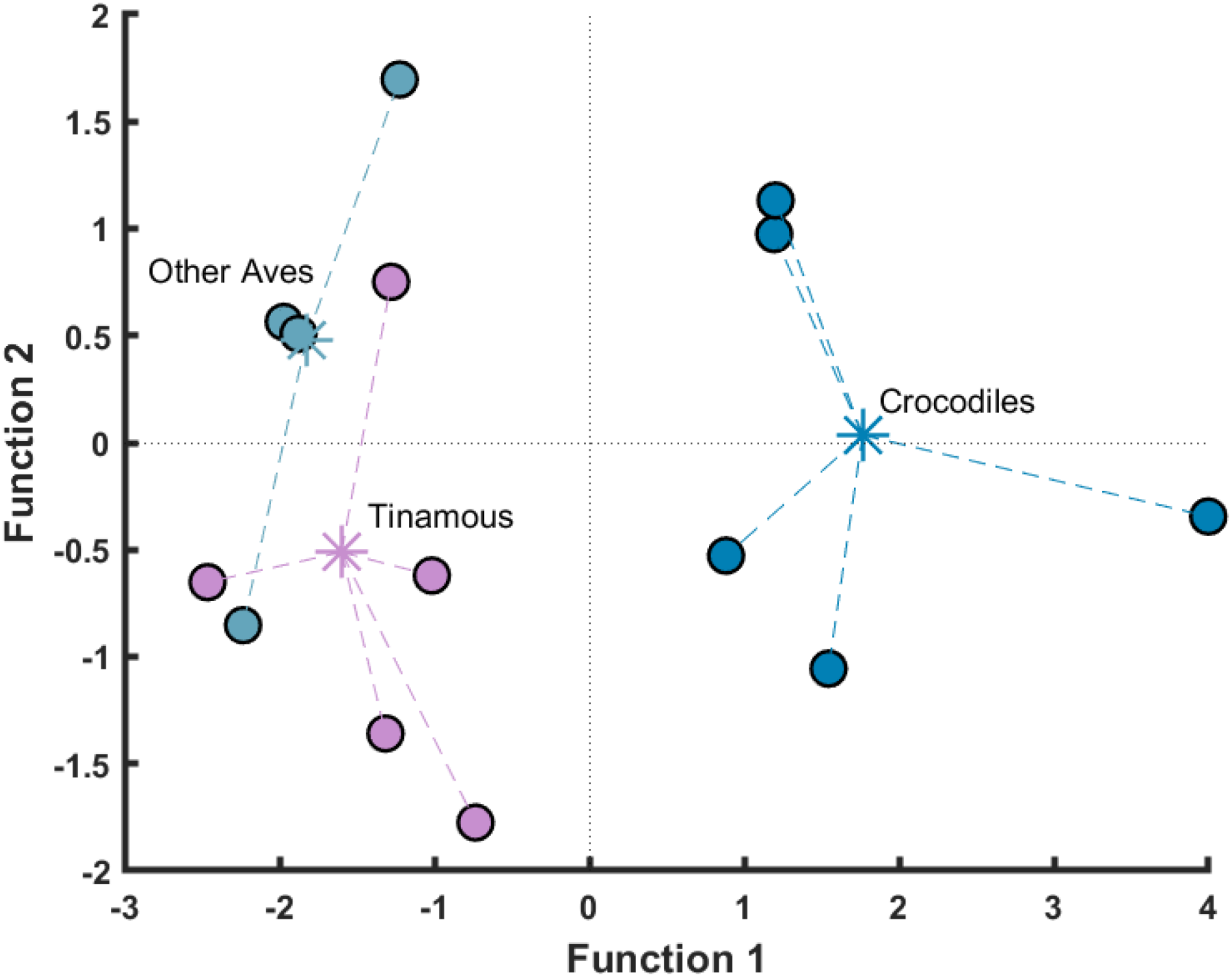
Results of the Discriminant Function Analysis (DFA), indicating that the crocodile muscles were clearly identified, whereas the tinamous and other avian species had somewhat more problematic classifications. Group centroids (the mean discriminant scores) are represented as “*” symbols, which represent the relative Mahalanobis distance between each case and the group centroid.

We were mostly successful in correctly predicting the species assignment from the PCSA:AA of each muscle using a DFA, correctly classifying 86.7% of all PCSA:AAs with their corresponding species (Figure 6). All crocodile and ‘other Aves’ muscles were correctly classified (100%). 66.7% of tinamou muscles were correctly classified, with 33.3% of tinamou muscle data incorrectly classified as other avian species (turkey, emu, chicken or ostrich; data grouped). No Aves data were incorrectly classified as belonging to crocodiles, with a clear separation of Crocodylia and Aves samples.

### 3.6 MANOVA and Games–Howell post hoc test results

The IT2/AIL, IT3/PIL, FMTE/FMTL, FMTI/(FMTIM+FMTM), ILFB, IF/IFE, ADD1/PIFM and GI/(GIM + GM) PCSA:AA ratios were comparably similar between all crocodiles and birds (*p* ≥ 0.05, within a 95% confidence interval). In contrast, the IT1/IC, FTI3/FCM, ISTR/ISF, CFB/CFP, FL, TA/EDL and FHL/FDL (lower limb muscle homologies following Crocodylia/Aves pattern as per Hattori & Tsuihiji, 2021) PCSA:AAs were all significantly distinguishable between crocodiles and birds (*p* ≤ 0.05, within a 95% confidence interval; Table 4). Of these muscles, the IT1/IC, TA/EDL and FHL/FDL were statistically distinguishable between all groups, indicating that these particular muscles' PCSA:AAs are species‐specific, and do not share a common PCSA:AA with other archosaurs. Therefore, the PCSAs of these three muscles cannot be easily predicted from the AA in fossil specimens using an ‘all‐archosaur’ approach. Those other muscles which were statistically different between Crocodylia and Aves (Table 4) should not have the PCSA of the respective muscle estimated from the AA using the same method. Rather, crocodile‐specific and Aves‐specific methods must be adopted, supported by the DFA. Contrastingly, eight muscles' PCSA:AA ratios (IT2/AIL, IT3/PIL, FMTE/FMTL, FMTI/(FMTM+FMTIM), ILFB, IF/(ITC + IFE), ADD1/PIFM and GI/(GM + GIM)) were statistically similar between crocodiles and Aves, suggesting that it is possible to predict each muscles' PCSA from their respective AA in all archosaurs. This is also consistent with the inference that PCSA can be predicted from AA in crown group Aves with some accuracy for these muscles.

**TABLE 4:** Results of the MANOVA and Games Howell post hoc tests, in which significance levels are reported if the mean difference is significantly different within a 95% confidence interval

Games–Howell post‐hoc test			Mean difference	SE	*p*	95% confidence interval	
Muscle	Between‐groups variability					Lower bound	Upper bound
IT1/IC	Crocodile	Tinamou	0.917	0.237	**0.020**	0.188	1.646
		Other Aves	1.258	0.214	**0.008**	0.506	2.010
	Tinamou	Crocodile	−0.917	0.237	**0.020**	−1.646	−0.188
		Other Aves	0.340	0.108	**0.049**	0.002	0.679
IT2/AIL	Crocodile	Tinamou	6.077	1.936	0.073	−0.824	12.978
		Other Aves	−0.411	4.592	0.996	−16.281	15.458
	Tinamou	Crocodile	−6.077	1.936	0.073	−12.978	0.824
		Other Aves	−6.488	4.164	0.385	−23.889	10.912
IT3/PIL	Crocodile	Tinamou	7.735	3.490	0.182	−4.699	20.169
		Other Aves	7.477	3.500	0.196	−4.927	19.881
	Tinamou	Crocodile	−7.735	3.490	0.182	−20.169	4.699
		Other Aves	−0.258	0.286	0.671	−1.357	0.841
FMTE/FMTL	Crocodile	Tinamou	1.075	0.368	0.086	−0.209	2.358
		Other Aves	0.846	0.379	0.164	−0.418	2.109
	Tinamou	Crocodile	−1.075	0.368	0.086	−2.358	0.209
		Other Aves	−0.229	0.122	0.246	−0.638	0.180
FMTI/(FMTM+FMTIM)	Crocodile	Tinamou	−0.142	0.102	0.405	−0.460	0.176
		Other Aves	−0.186	0.190	0.621	−0.816	0.443
	Tinamou	Crocodile	0.142	0.102	0.405	−0.176	0.460
		Other Aves	−0.045	0.172	0.964	−0.711	0.621
ILFB	Crocodile	Tinamou	0.592	0.462	0.461	−0.892	2.076
		Other Aves	0.303	1.077	0.958	−3.474	4.081
	Tinamou	Crocodile	−0.592	0.462	0.461	−2.076	0.892
		Other Aves	−0.288	1.001	0.956	−4.326	3.749
IF/(IFE+ITC)	Crocodile	Tinamou	1.368	0.559	0.141	−0.598	3.334
		Other Aves	0.788	0.601	0.446	−1.130	2.705
	Tinamou	Crocodile	−1.368	0.559	0.141	−3.334	0.598
		Other Aves	−0.580	0.240	0.162	−1.493	0.333
FTI3/FCM	Crocodile	Tinamou	9.406	2.183	**0.027**	1.626	17.186
		Other Aves	9.177	2.218	**0.028**	1.489	16.865
	Tinamou	Crocodile	−9.406	2.183	**0.027**	−17.186	−1.626
		Other Aves	−0.229	0.393	0.839	−1.872	1.414
ISTR/ISF	Crocodile	Tinamou	0.541	0.088	0.004	0.251	0.831
		Other Aves	0.295	0.141	0.174	−0.141	0.731
	Tinamou	Crocodile	−0.541	0.088	**0.004**	−0.831	−0.251
		Other Aves	−0.246	0.117	0.222	−0.702	0.210
ADD1/PIFM	Crocodile	Tinamou	0.216	0.095	0.111	−0.049	0.481
		Other Aves	0.441	0.207	0.205	−0.323	1.206
	Tinamou	Crocodile	−0.216	0.095	0.111	−0.481	0.049
		Other Aves	0.226	0.207	0.571	−0.538	0.989
CFB/CFP	Crocodile	Tinamou	3.073	0.751	**0.030**	0.444	5.702
		Other Aves	3.263	0.757	**0.024**	0.648	5.879
	Tinamou	Crocodile	−3.073	0.751	**0.030**	−5.702	−0.444
		Other Aves	0.191	0.176	0.556	−0.345	0.726
GI/(GIM + GM)	Crocodile	Tinamou	−0.084	0.483	0.984	−1.794	1.626
		Other Aves	−3.858	1.370	0.103	−8.832	1.116
	Tinamou	Crocodile	0.084	0.483	0.984	−1.626	1.794
		Other Aves	−3.774	1.283	0.118	−9.130	1.582
FL	Crocodile	Tinamou	−3.287	0.464	0.001	−4.736	−1.839
		Other Aves	−0.335	0.767	0.903	−3.448	2.777
	Tinamou	Crocodile	3.287	0.464	**0.001**	1.839	4.736
		Other Aves	2.952	0.881	**0.044**	0.104	5.799
FHL/FDL	Crocodile	Tinamou	8.782	2.000	**0.025**	1.653	15.911
		Other Aves	7.758	2.002	**0.038**	0.633	14.884
	Tinamou	Crocodile	−8.782	2.000	**0.025**	−15.911	−1.653
		Other Aves	−1.023	0.073	**0.001**	−1.317	−0.730
TA/EDL	Crocodile	Tinamou	0.917	0.237	**0.020**	0.188	1.646
		Other Aves	1.258	0.214	**0.008**	0.506	2.010
	Tinamou	Crocodile	−0.917	0.237	**0.020**	−1.646	−0.188
		Other Aves	0.340	0.108	**0.049**	0.002	0.679

Abbreviations: *p*, value for significance test; SE, standard error.

Bold values represent significant *p* values < 0.05.

### 3.7 Which muscles can have PCSA estimated from AA and which equation is best to use?

**FIGURE 7:**
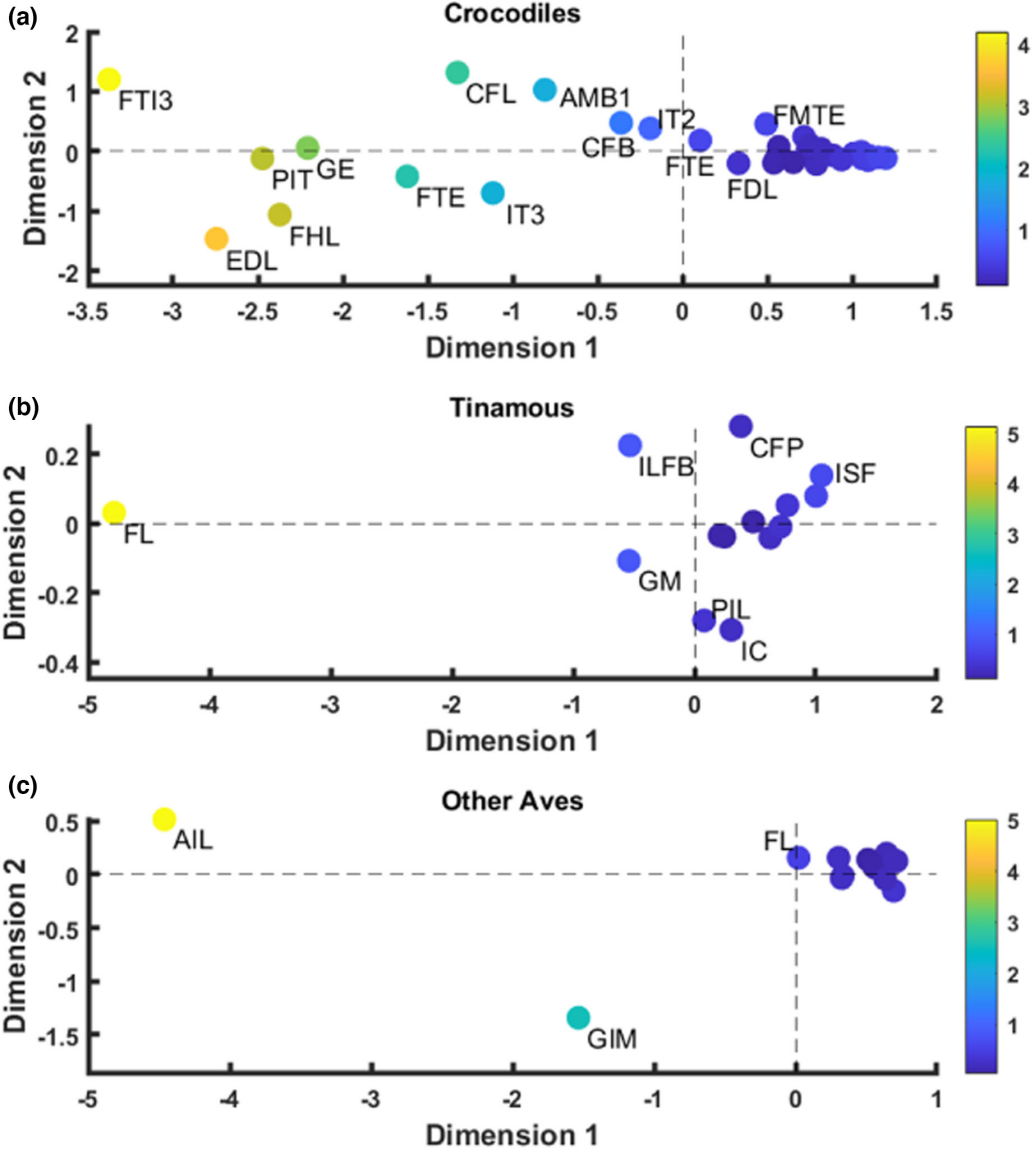
Visual representation of the multidimensional scaling using a Euclidean distance model with a derived stimulus configuration on all muscle PCSA:AA. Data for crocodiles (Stress = 0.0276; *R* ^2^ = 0.998), tinamous (Stress = 0.012; *R* ^2^ = 0.999) and other Aves (Stress = 0.054; *R* ^2^ = 0.996), using Kruskal's algorithm (Kruskal, 1964). Those muscles which plotted closer to the derived mean are in blue on the colour scale, whereas those muscles which plotted further from the derived mean are coloured yellow/green accordingly with the coloured scale bar. Only muscles which plotted furthest from the clusters were labelled; these were the outliers. All other muscle names (those within the clusters) can be found in Table 2.

Our results (Figure 7) aligned well with those from the MANOVA and Games‐Howell Post‐Hoc tests, with two exceptions: the GIM (Aves only) and IT2/AIL (crocodiles and ‘other Aves’ only). Upon inspection of the raw input data for these two muscles, the variability visualised in Figure 7 could be explained by the ‘other Aves’ data driving the discrepancy, in which the PCSA:AA ratio was much larger (in both circumstances) and more variable within species in the ‘other Aves’ group, than in the crocodile and tinamou (the latter of which lacked GIM data) samples. Therefore, the GIM/GI and IT2/AIL muscles needed to be added to our list of muscles which did not fit within a ‘one size fits all' analysis.

Additionally, intra‐specific prediction of PCSA from AA measurements was further divided into a ‘one size fits all’ prediction versus a muscle‐specific prediction, dependent upon those PCSA:AAs identified as outliers in the MDS. All muscles grouped in the cluster (i.e. those in dark blue in Figure 7) were assigned to the ‘one size fits all’ approach, meaning that a generic prediction model (Equation 2) could be generated. Figure S2 shows linear regression (logged) results. All ‘outlier’ muscle data were treated as independent values, and assigned an individual, unique approach for estimating muscle‐specific PCSA from AA.

### 3.8 Can we predict PCSA from AA in extant archosaurs?

Due to levels of non‐significant differences between our results for the tinamous and crocodiles, despite the muscles being discriminately differential (DFA), we infer that it is possible to predict PCSA from AA using the same prediction model for both the crocodiles and tinamous in the following six muscles: IT3/PIL, FMTE/FMTL, FMTI/(FMTIM+FMTM), ILFB, IF/IFE and ADD1/PIFM. Additionally, we can include all ten major muscles (IF/ITC, PIFI1/IFI, PIFI2/[ITM + ITCR], ADD2/PIFL, FTI1, PIFE1, PIFE2/OM, PIFE3, TA/EDL and FB) which fell within the clusters identified in Figure 7. To make this prediction for a total of 16 main muscles, we computed a ‘one size fits all’ linear regression model (Figure S2C) that estimated PCSA from the measured AA in our sample, using the following equation (raw PCSA units are in m^2^; AA in mm^2^):

(3) $$\text{PCSA}=\left(3\times 10^{-7}\left(\mathrm{AA}\right)+5\times 10^{-5}\right)$$

Unique, independent prediction equations should instead be used for the following nine muscles: IT1/IC, IT2/AIL, FTI3/FCM, ISTR/ISF, CFB/CFP, GI/(GM + GIM), FHL/FDL, EDL/TC and FL. From the MDS, we identified a further six muscles which were not located within the ‘cluster’, which we identified as ‘outliers’; those which did not fit into the ‘one size fits all’ approach. We, therefore, added unique prediction equations for those six muscles (AMB1 [of Crocodylia], FTI2, FTE/FCLP, PIT, CFL [insertion AA], GE/GL) that were not included in statistical analyses due to insufficient comparative sample sizes (Figure 7). Using the two approaches for estimating PCSA from AAs in crocodiles and tinamous, we computed a series of linear regressions (Table 5). Using the equations to predict PCSA, we found that 71.7% of archosaur PCSAs were predicted from AAs within <5% of their original measured value (Table 6) and 92.5% of PCSAs were predicted within <10% of their dissected measurement. We also found that using this same equation‐based approach for our ostrich specimen obtained less accuracy, but still 71.4% within <10% of PCSA measurements (Table 6).

**TABLE 5:** Prediction equations for archosaurian hindlimb muscle PCSA:AAs. 15 unique linear regression equations, followed by the ‘one size fits all’ (Equation 3) approach applicable to all ~16 other major hindlimb muscles

Muscle	Prediction equation	SE	ME (95% confidence)	Use for:	
				Crocodiles	Birds
IT1/IC	PCSA = 0.2686(AA) + 16.986	20.01	±48.978	✓	✓
IT2/AIL	PCSA = −0.0329(AA) + 59.337	12.92	±51.896	✓	✓
AMB(1)	PCSA = 14.515(AA)−109.75	2.60	±11.197	✓	
FTI2	PCSA = 4.0948(AA)−4.7909	1.91	±8.229	✓	
FTI3/FCM	PCSA = 1.1953(AA) + 31.675	12.27	±52.789	✓	✓
FTE/FCLP	PCSA = 0.8314(AA) + 65.048	31.96	±136.369	✓	✓
PIT	PCSA = 0.901(AA) + 25.696	4.96	±21.354	✓	
CFB/CFP	PCSA = 0.0689(AA) +80.195	44.7	±142.277	✓	✓
CFL (insertion AA)	PCSA = 17.356 (AA)−325.13	1.83	±7.908	✓	
ISTR/ISF	PCSA = −0.1193(AA) + 80.123	0.71	±1.72629	✓	✓
GE/GL	PCSA = −0.8186(AA) + 124.49	18.11	±57.659	✓	✓
GI/(GM + GIM)	PCSA = 1.3178(AA)−8.052	7.67	±18.756	✓	✓
EDL/TA	PCSA = 0.7329(AA) + 40.727	10.21	±43.948	✓	✓
FHL/FDL	PCSA = 2.6843(AA) + 21.412	5.27	±22.667	✓	
FL	PCSA = −0.2664(AA) + 95.42	27.90	±71.714	✓	✓
All other muscles (Equation 3)	PCSA = 0.2826(AA) + 40.24	7.97	±15.948	✓	✓

*Note*: Units are in mm^2^. The ‘Use for’ columns indicate which data were used in each equation (from our MANOVA/Games–Howell and MDS tests).

Abbreviation: SE, standard error.

**TABLE 6:** Likelihood tests of predicted vs. measured PCSA. Unique (individual linear regression) predictions for 80 (or 76.9% of all) muscle samples fell within <5% of the measured value

Unique prediction equation			One size fits all approach	
Range (%)	Count	% of muscles	Count	of muscles
>5	80	76.92	38	71.7
5–10	15	14.42	11	20.8
10–20	5	4.81	5	7.55
20–30	2	1.92	0	0
30–40	1	0.962	0	0
40–50	1	0.962	0	0

Ostrich equations		
Range (%)	Count	% of muscles
>5	1	7.14
5–10	9	64.3
10–20	0	0
20–30	0	0
30–40	1	7.14
40–50	2	14.3
50–60	1	7.14

*Note*: Average percentage differences between absolute values of predicted and measured values were 4.30% (median = 2.54%). ‘One size fits all’ (Equation 3) predictions for 38 (or 71.7% of all) muscle samples fell within <5% of the measured value. Average percentage differences between these predicted and measured values were 3.96% (median = 2.73%). Below: Ostrich equation = predictions from equations applied to 14 muscles from the ostrich specimen's measured PCSAs. Ostrich muscle prediction differences >39% = IC (unique equation), FMTL, FMTIM and ILFB (using the one size fits all equation).

### 3.9 What PCSA estimates do we obtain for *Coelophysis bauri* from AA reconstructions?

**FIGURE 8:**
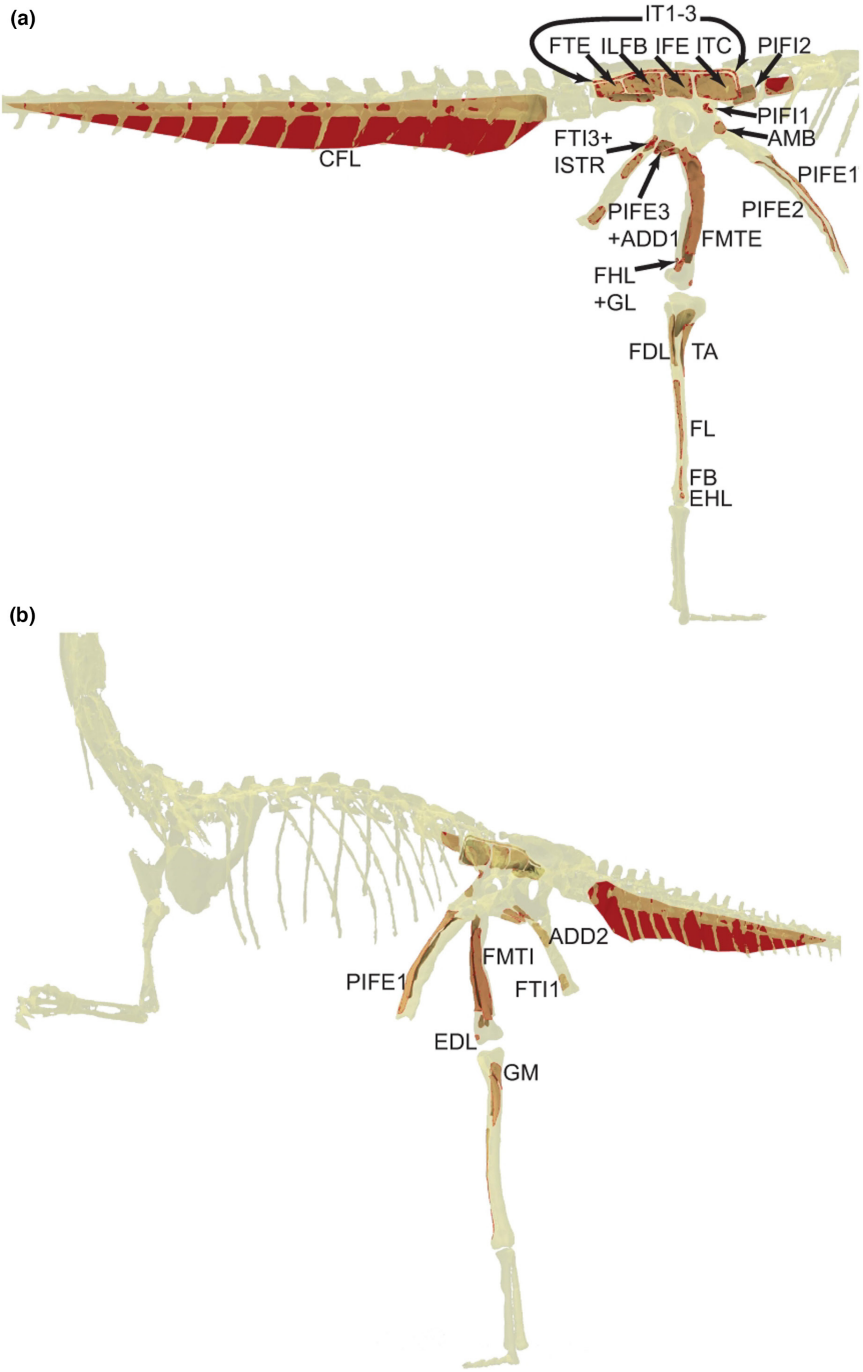
Muscle origin AAs (in red) reconstructed on *Coelophysis bauri* following Bishop, Cuff, et al. (2021); executed in Rhinoceros software. (a) Right lateral view (muscles having mostly lateral/caudal/cranial origins); (b) Left oblique craniomedial view (only GM and FMTI have truly medial origins; others in B have cranial/lateral origins). The AA of the CFL origin as shown reflects its origin primarily from caudal vertebral centra and haemal arches (chevrons); but some origin would be from intermediary fascia or other soft tissue, which studies to date have not explicitly separated from bony origins.

We were able to estimate the PCSAs for hindlimb muscles from the digitised muscle origin AAs (Table 7) in the model of the theropod dinosaur *Coelophysis bauri* (Figure 8). When compared with variant 3 from Bishop, Cuff, et al. (2021), we found that only three (FTE, CFL, FHL) of 31 muscles had ratios of estimated PCSA values between the two studies' approaches within 0.50–1.5 (estimated here vs. variant 3) (mean ratio 0.31, S.D. 0.57). Our four largest muscles (greatest PCSA estimates), were (in order from smaller to larger) the GI, FMTE, CFL and AMB1 here; in contrast to the larger PIFE3, ISTR, FB and IFE in Bishop, Cuff, et al. (2021). Compared with the total muscle masses and PCSAs predicted from scaling data on six extant bipeds from Bishop, Wright, et al. (2021), variant 3 of Bishop, Cuff, et al. (2021) did well at predicting muscle mass (ratio of predicted mass/mass from variant 3 = 1.01) but overestimated PCSA (ratio 0.424). In contrast, our method underestimated total hindlimb muscle PCSA (ratio 2.7) versus the same scaling data.

**TABLE 7:** Predicted PCSA values for 30 *Coelophysis* hindlimb muscles using the approaches developed in this study. Muscle acronyms follow Crocodylia names (and homologies) in Table 2; except for IFE and ITC of Aves. IT1‐IT3 AA was continuous and split evenly between all three heads

Muscle	Equation used	AA (mm^2^)	Estimated PCSA (mm^2^)	PCSA variant 3—Bishop, Cuff, et al. (2021)	Ratio of Estimated PCSA to variant 3
IT1	Unique	109.84	46.49	420.07	0.11
IT2	Unique	109.84	55.72	772.72	0.07
IT3	OSFA	109.84	69.74	1006.81	0.07
FMTE	OSFA	1792.98	521.84	1507.59	0.26
FMTI	OSFA	1145.29	347.86	1527.91	0.23
AMB	Unique	131.20	1794.67	584.93	3.07
ITC	OSFA	1003.66	309.82	2963.88	0.10
IFE	OSFA	670.83	220.43	8981.45	0.02
PIFI1	OSFA	64.06	57.45	1231.13	0.05
PIFI2	OSFA	750.94	241.94	1332.81	0.18
ILFB	OSFA	740.95	239.26	683.19	0.35
FTI1	OSFA	207.26	95.91	359.12	0.27
FTI3	Unique	73.25	119.23	651.69	0.18
FTE	Unique	363.07	366.91	546.11	0.67
ADD1	OSFA	143.38	78.75	2049.76	0.04
ADD2	OSFA	250.96	107.65	828.71	0.13
PIFE1	OSFA	1191.01	360.15	1199.15	0.30
PIFE2	OSFA	851.50	268.96	1244.34	0.26
PIFE3	OSFA	222.47	99.99	3314.54	0.03
ISTR	Unique	111.80	66.79	5269.76	0.01
CFB	Unique	1170.75	160.86	1506.53	0.11
CFL^a^	Unique	107.03	1532.48	1191.15	1.29
GI	Unique	340.87	441.14	1119.44	0.39
GE	Unique	87.06	53.22	1075.59	0.05
EDL	Unique	41.44	71.10	275.03	0.26
FHL	Unique	68.09	204.19	369.74	0.55
EHL^b^	OSFA	23.83	46.64	2567.96	0.02
TA	OSFA	590.12	198.75	984.17	0.20
FDL	OSFA	527.50	181.93	591.76	0.10
FL	Unique	296.57	16.41	1758.48	0.01
FB	OSFA	72.91	59.82	5995.40	0.01

*Note*: Equation used: ‘Unique’ = individual/muscle‐specific; ‘OSFA’ = ‘One size fits all’ (Equation 3). Predicted PCSA values are also provided (see Methods). These predicted values are compared against the values from ‘variant 3’ in Bishop, Cuff, et al. (2021).

^a^ Note CFL PCSA was estimated from insertion AA, not origin AA.

^b^ Note the EHL muscle did not have sufficient data in our extant sample and its estimation here is for completeness and comparison; usage of equation 3 for muscles outside our dataset is debatable (see Discussion). IOC and PP data from Crocodylia were not estimated (see Hutchinson (2002) and Bishop, Cuff, et al. (2021) for justification).

## 4 DISCUSSION

We found varied levels of support for estimating PCSA from AA in extant archosaurs. This estimation method may be useful for predicting PCSA in extinct archosaurs, and complements other estimation methods such as scaling and volumetric reconstruction. There was certainly variation and error in our dataset (e.g., Figure S2) that might have confounded some of the analyses, but we found consistent measurement of muscle fascicle lengths for our Nile crocodiles and tinamous; key data for quantifying PCSA. Our analyses of areas of known geometry (Table S2) showed that, under the best case, areas could be digitised with high accuracy. However, unless the meshes created by our Delaunay triangulation method were edited (as we normally did; but did not for the known geometry) prior to area quantification, complex shapes could have much more inaccurate (overestimated in both our cases) areas. Next, our crocodile AA analysis (Table S1) revealed moderately high variation, so estimates of PCSA from AA inevitably would suffer from some error due to methodological ‘noise’. This error is likely to primarily be from difficulties digitising complex 3D muscle AAs on bones, with slippery surfaces and boundaries that can be difficult to discern; and not very high resolution in terms of number of points digitised. Finally, applying our predictive equations for PCSA from AA to our 65.3 kg ostrich specimen showed strong predictive ability for >70% of muscles; yet some muscles could be more poorly predicted (39% + error; Table 6). This suggests that larger‐bodied taxa can still have reasonable PCSA values predicted using our method. However, our comparison with Bishop, Wright, et al. (2021) scaling data (six extant bipeds) for muscle PCSA showed that our method substantially underestimates total PCSA compared with those data, suggesting that (archosaur hindlimb) AA scales with negative allometry, which to our knowledge has not been investigated in extant tetrapods and deserves focus.

Our PCA analyses of the more complete crocodile sample showed that there were grossly similar PCSA:AA ratios across our subjects and muscles, with no strong patterns across the hindlimb muscles despite diverse architecture, positions and actions. It is still worth exploring that possibility, however, in future studies, as we did notice hints of possibly meaningful PCSA:AA trends in our average raw data (Table S1). Our between‐groups PCA showed substantial variability and overlap between archosaurian groups, but our DFA revealed that we were able to obtain good categorizations of our muscle samples, with no avian muscles being misassigned as crocodylian. Furthermore, our quantitative analyses with MANOVA and Games‐Howell post hoc tests demonstrated that there were some muscles with similar PCSA:AA ratios in archosaurs (Figure S2), whereas some muscles had unique ratios requiring independent analyses rather than a ‘one size fits all’ approach. These patterns ultimately led to statistical justification for 15 muscles using the former approach vs. 16 using the latter, and a reasonable case for estimating the PCSA of crocodile CFL muscles using insertion AA. Importantly, these predictive equations had strong accuracy (<5–10% error) in predicting PCSA from AA in our archosaur muscles, bolstering confidence in their usage for archosaurian hindlimbs.

We then applied our methods to the dinosaur *Coelophysis*, finding that estimates of maximal isometric muscle forces were generally considerably lower than estimated by Bishop, Cuff, et al. (2021), except that we obtained, in particular, large force capacity for M. caudofemoralis longus (CFL) which is much more plausible because of the obvious large size of the CFL (see also Demuth et al., 2022; Hutchinson et al., 2011; Persons & Currie, 2011). The differences between these two studies' results raise the question, which of these methods is better for estimating hindlimb muscle force‐generating capacity in Archosauria? Both are data‐driven; even by roughly similar data (e.g. PCSAs from overlapping samples of extant Archosauria; calculated essentially identically). Bishop, Cuff, et al. (2021) ‘variant 3’ draws more on musculotendon unit lengths (from musculoskeletal modelling of the fossil taxon) as input data, whereas our method relies on AA estimation for fossils. It is perhaps likely that one method is superior for certain muscles in certain taxa but another for other muscles and taxa; this needs further investigation.

A concern might arise about autocorrelation or circularity in using extant taxa to reconstruct AAs in extinct taxa. Muscle AAs are ultimately based both on phylogenetic inference (the EPB) and direct observation of muscle scarring (see Methods above); much as they have been for the history of reconstruction of musculature in extinct taxa (e.g. Gregory & Camp, 1918; Romer, 1923a, 1923b, 1923c, 1927). Importantly, the phylogenetic inference is not a simple ‘transferral’ of AAs from extant taxa to extinct taxa—we recognise the variations and nuances present in the fossil anatomy, framed within the context of phylogeny via reciprocal illumination. Indeed, this recognition and illumination is vital when we encounter morphologies unique to the fossil record. In all cases, we use the fossil anatomy as much as possible to guide recognition of (a) the locus of each attachment and (b) the size/shape of said attachment. In doing so, this helps minimise the ‘everyarchosaur’ issue (Bishop, Cuff, et al. 2021). No direct osteological correlate may indeed be preserved in some cases, but a *Coelophysis* ilium is differently sized/shaped to that of a bird or crocodylian, and thus estimates of its AA are partly independent of the EPB, and not simply autocorrelation or circularity. Volumetric reconstructions of muscle sizes proceed via similar methods and their assumptions similarly are not autocorrelation or circularity.

Application of empirical muscle architecture vs. body mass scaling data from Bishop, Wright, et al. (2021) revealed that total muscle PCSA was not well predicted by variant 3 compared with that scaling equation (~236% overestimate by variant 3). This raises the question of whether the previous scaling data presented by Bishop, Wright, et al. (2021) in which only two extant obligate striding bipedal clades (birds and humans), out of a total of six species, are insufficiently reliable for predicting muscle architecture. Alternatively, perhaps variant 3 is more reliable, or even a combination of both. Our method's relative success at predicting an ostrich's PCSA suggests that it is not allometry causing the discordance between our results presented here and that of variant 3's; but rather that allometry might be the cause of the discordance between the data presented previously by Bishop, Wright, et al. (2021) and variant 3 which was implemented here. The question of whether our AA:PCSA approach or that of variant 3 is more reliable hinges on answering that question as well. We feel that this is unanswered, and that further experimentation is required with larger datasets.

In this study, we asked how different methods for estimating muscle PCSA and mass compare in terms of advantages and disadvantages. The answer is a nuanced one: those estimating muscle PCSA or *F*_max_ (or mass) for extinct archosaurs using such approaches should compare results and weigh the plausibility, objectivity and other merits on a case‐by‐case basis. These comparisons could be done in light of what extant study taxa the underlying sample came from, their body size range vs. the fossil taxon's, potential syn−/autapomorphies with/relative to crocodiles or birds (vs. the ‘everyarchosaur’ problem noted by Bishop, Cuff, et al., 2021), and potential errors involved in all data sources and methods. However, there is cause for some optimism because there are some consistent patterns of muscle architecture and skeletal morphology across extant archosaurs. Such optimism is bolstered by the independent approach devised by Demuth et al. (2022), successfully using volumetric polygonal mesh models to estimate muscle masses in both extant and extinct archosaurs (i.e. from which PCSA could be calculated using other approaches). Hence there are multiple methods available that could cope with some of the above‐mentioned problems. 3D imaging methods (CT and MRI scanning, etc.) applied to extant taxa offer promise but also can have constraints in terms of what kinds of entheses are visible and whether sufficient resolution of attachments is feasible, which should be considered in applying them. Regardless, small sample sizes, our emphasis on muscle origin AA, taphonomic distortion of fossils and fundamental differences in gross appendicular morphology across extant and extinct archosaurs are problems common to most if not all of these methods. Furthermore, digital volumetric and some other methods give estimates of muscle mass, not PCSA and thus do not directly inform on muscle *F*_max_. To do so, they require estimates of primarily muscle fascicle (or fibre) lengths (see Equation 1; pennation angle is less vital but also relevant). Yet musculoskeletal models and simulations, which use such *F*_max_ data, typically require muscle fascicle length estimates anyway; our PCSA:AA method does not circumvent that limitation. Relationships of muscle mass or PCSA with fascicle lengths in extant saurians are highly variable across muscles and can be variable within muscles (see Bates & Schachner, 2012; Bishop, Cuff, et al., 2021), so this is a significant challenge (also see Charles et al., 2022).

Additionally, certain major differences between PCSAs (or masses) and AAs in crocodiles and birds are easily attributable to identifiable evolutionary divergences (e.g. M. caudofemoralis longus reduction in birds vs. ancestral Sauria; Gatesy, 1990) that reconstructions of fossil archosaurs can then account for quantitatively (e.g. Allen et al., 2013; this study). In the case of the CFL muscle, Allen et al. (2013) reconstructed its mass in the same skeletal model of *Coelophysis* as ~0.541 kg (body mass estimate: 18.3 kg) using a volumetric approach (Hutchinson et al., 2011; Persons & Currie, 2011). Our PCSA of 1530 mm^2^ estimated from CFL insertion AA would correspond (using Equation 1) to a mass of ~0.200 kg (body mass estimate from Bishop, Cuff, et al. (2021): 13.1 kg) if a fibre length of 0.123 m and pennation angle of 0° were assumed (Bishop, Cuff, et al., 2021). Thus, scaling by the two models' 0.716 ratio of body mass, our CFL mass would be 36% of their estimate from an independent digitally volumetric approach, so in comparison about half that expected for our model's mass. Again, which CFL mass estimate is ‘superior’? Our current approach could be considered more data‐driven in terms of abundance of data from extant taxa (e.g. to date, four non‐avian saurian species and specimens for CFL volumetric estimates; variable amounts for other muscles and methods – e.g. see Bates & Schachner, 2012; Bishop, Cuff, et al., 2021). Digital volumetric estimation of CFL muscle masses for extant non‐avian Sauria, which draws on a priori understanding of muscle AA, appears to be reasonably accurate (within ~5% error; Hutchinson et al., 2011; Persons & Currie, 2011).

In recent years there has indeed been a major push from not just identifying muscle attachments for extinct animals, but quantifying muscle sizes and forces driven primarily by enhanced computer modelling of feeding and locomotor behaviours (e.g. Bishop, Cuff, et al., 2021; Bishop, Falisse, et al., 2021; Bishop, Michel, et al., 2021; Gignac & Erickson, 2017; Herbst et al., 2022; Hutchinson, 2002; Snively et al., 2013; Snively & Russell, 2007). There remains plenty of room for improvement and innovation, as echoed by similar recent studies (Bates et al., 2021; Broyde et al., 2021; Rhodes et al., 2021). Such progress remains dependent in part upon more data for extant taxa, as provided here; any reliable data should be informative and help drive progress.

Here we have answered the call to ‘go back to the bones’ by Bates et al. (2021) in multiple ways: (1) by studying multiple specimens per species rather than one, (2) by compiling a comprehensive sample of (hindlimb) muscles allowing assessment of variation, (3) by testing how consistently muscle fascicle lengths that help determine PCSA are, (4) through a multi‐stage statistical analysis that objectively and rigorously explores the relationships of PCSA and AA, and (5) by showing with statistical tests that our sample's estimates of PCSA from AA, in general, are reasonably accurate. This gave us some confidence in estimating PCSA with our new approach for the fossil archosaur *Coelophysis*. Yet one cannot go ‘back to the bones’ without remaining firmly grounded in data from muscles. We expand on the call by Bates et al. (2021) by urging (as their data also hint) that there may not be universal principles relating PCSA to AA (or PCSA from volumetric estimates). Findings from rodent jaws may not relate well to those from archosaur hindlimbs and vice versa, so clade‐specific foci and phylogenetically informed studies are important to explore what variation exists in nature. We do advise caution though, modern archosaurs represent but a small subsample of their former diversity, and our investigation was only able to sample a few species, which were in turn represented by individuals with small body masses, and with considerable variation in PCSA versus AA relationships (Figure S2). The predictions made here may not scale into the largest archosaurian species, and broad comparative studies of archosaur hindlimb muscle area estimation (e.g. Demuth et al., 2022; Rhodes et al., 2021; Snively et al., 2019), and calculations using those data, will need reinvestigation with improved data on PCSA:AA (or volumetric) relationships.

However, there are more ways forward in this area of inquiry. One idea for further investigation is quantitative study of three‐dimensional muscle shape: perhaps the relationships of muscle architecture with skeletal geometry hinges in part on that aspect of muscular geometry. Another question, tentatively touched on here, is whether PCSA and AA are sometimes more correlated when muscles have fleshy (periosteal) attachments to bones. The data present here and in past studies also raise the fascinating question of how PCSA:AA ratios evolve, and how they are determined by more proximate and ultimate factors in development, mechanics and geometry (e.g. ‘packing’ into three‐dimensional organisms; or body contouring). What might constrain or canalize these ratios and thereby influence their evolution? It is an exciting time to ask these questions, and to push forward means to apply their answers to major palaeobiological mysteries and evolutionary transitions.

## AUTHOR CONTRIBUTIONS

JRH: Devised the study; PJB: Wrote MATLAB code; ARC: Collected *Crocodylus* AA data; KM, JRH: Collected *Eudromia* AA data; ARC: Collected *Crocodylus* PCSA data; JRH, PJB, KM: Collected *Eudromia* PCSA data; JRH: Collected additional avian data; JRH: Collected accuracy and repeatability tests with geometry and *Caiman* muscles; RG, KM, ARC, JRH: Analysed digitised AA data; PJB, JRH: Reconstructed *Coelophysis* AAs and estimated PCSAs; ALAW, ARC, JRH: Conducted statistical and final data analyses and interpretations; JRH: Wrote the main paper; ARC, ALAW, PJB: Contributed substantially to paper writing. All authors approved the final manuscript.

## CONFLICT OF INTEREST

The authors declare no conflict of interest.

## Supporting information


Appendix S1

## Data Availability

Necessary code and data are provided on Figshare at: https://figshare.com/projects/Anatomically_Grounded_Estimation_of_Hindlimb_Muscle_Sizes_in_Archosauria/144135
