# Supplementary material for: Anatomically grounded estimation of hindlimb muscle sizes in Archosauria
Source: J Anat. 2022 Oct 7;242(2):289–311. doi: 10.1111/joa.13767 (PMC9877486; doi:10.1111/joa.13767)

SUPPLEMENTARY INFORMATION

**Table S1.** Ratios of PCSA/AA and direct/indirect attachment categorisation for tinamou, “other Aves” and Nile crocodile hindlimb muscle origins. See Table 2 for abbreviations and homologies. Not all muscles are discussed in this study (e.g. avian M. plantaris (PL) and digital flexors FPD2-4, FP&PD2+3; crocodile M. pubotibialis internus (PIT)). D/I = direct (D; fleshy muscle)/indirect (I; tendon/aponeurosis) attachments. “Mean ratio croc/tinamou” shows crocodile vs. tinamou ratios, with those 0.5-1.5 in bold. Proximal muscles (see Results) are those from IC/IT down to OL/PIFE3; following rows are distal muscles.

| **Tinamou**  **Muscle** | **Mean ratio- PCSA/AA** | **Mean ratio-other Aves** | **D/I** | **Crocodile Muscle** | **Mean ratio- PCSA/AA** | **D/I** | **Mean ratio- croc/tinamou** |
| --- | --- | --- | --- | --- | --- | --- | --- |
| IC | 0.72 | 0.43 | D | IT1 | 1.68 | I+D | 2.33 |
| AIL | 0.82 | 7.26 | I+D | IT2 | 6.85 | I+D | 8.31 |
| PIL | 0.91 | 1.18 | D | IT3 | 5.41 | I+D | 5.95 |
| AMB |  | 39.83 | D? | AMB1 | 13.22 | D |  |
| (AMB2) |  | 10.56 | D | AMB2 | 0.89 | D |  |
| FMTL | 0.38 | 0.49 | D | FMTE | 1.45 | D | 3.86 |
| FMTIM | 0.48 | 0.61 | D | FMTI | 0.35 | D | **0.73** |
| FMTM | 0.30 | 0.55 | D | - |  |  |  |
| ILFB | 1.26 | 1.62 | D | ILFB | 1.85 | D | **1.47** |
| IFE | 0.41 | 1.84 | D | IF | 1.81 | D | 4.39 |
| ITC | 0.44 | 0.76 | D | - |  |  | 4.13 |
| IFI | - |  | D | PIFI1 | 0.34 | D |  |
| ITCR |  | 6.20 | D | PIFI2 | 0.36 | D | **0.60** |
| ITM(+CR) | 0.59 | 1.01 | D | - |  |  |  |
| FCM | 0.74 | 1.10 | D | FTI3 | 10.77 | D | 13.62 |
| FCLP | 0.69 | 1.33 | D | FTE | 5.82 | D | 8.40 |
| FCLA |  | 6.89 | I | - |  |  |  |
| - |  |  |  | FTI1 | 1.19 | D |  |
| - |  |  |  | FTI2 | 3.65 | D |  |
| - |  |  |  | FTI4 |  | I |  |
| - |  |  |  | PIT | 8.43 | D |  |
| ISF | 0.18 | 0.42 | D | ISTR | 0.72 | D | 3.98 |
| PIFM | 0.80 | 0.59 | D | ADD1 | 1.71 | D | 2.12 |
| PIFL |  | 1.88 | D | ADD2 | 1.03 | D |  |
| CFP | 0.63 | 0.35 | D | CFB | 3.22 | D | 5.09 |
| OM | 0.09 |  | D? | PIFE2 | 0.18 | D | 1.93 |
| OL | n/a |  | D | PIFE1 | 0.17 | D |  |
| - |  |  |  | PIFE3 | 0.31 | D |  |
| GL | 3.82 | 4.85 | I+D | GE | 8.59 | I+D | 2.25 |
| GM | 1.35 | 3.09 | D | GI | 1.56 | D | **1.16** |
| GIM |  | 9.38 | I | - |  |  |  |
| PL |  | 1.15 | D | - |  |  |  |
| FL | 4.27 | 1.24 | D | FL | 0.98 | D | 0.23 |
| FB |  | 0.32 | D | FB | 1.88 | D |  |
| FDL | 0.22 | 1.32 | D | FHL | 9.00 | D | 41.26 |
| FHL |  |  | D | FDL | 1.79 | D |  |
| FPD2 |  |  | I+D | - |  |  |  |
| FPD3 |  | 33.18 | D | - |  |  |  |
| FPD4 |  | 71.48 | I+D | - |  |  |  |
| FP&PD2 |  |  | I+D | - |  |  |  |
| FP&PD3 |  | 0.28 | I+D | - |  |  |  |
| EDL | 0.21 | 0.23 | D | TA | 0.59 | D | 2.79 |
| TCt+f | 1.17 | 4.74 | I+D | EDL | 9.40 | I+D | 8.05 |
| - |  |  |  | IC | 0.67 | D |  |
| - |  |  |  | PP | 1.81 | D |  |

**Table S2.** Results from accuracy tests for digitising three areas of known geometry (Figure S1) and repeatability tests for three *Caiman* muscle origin AAs. All areas are in mm^2^. A1-A5 = values for areas measured. Mean = average of A1-A5; SD = standard deviation; Real A = actual known area (from Rhinoceros software). Points = number of points digitised.

| **Geometry** | **A1** | **A2** | **A3** | **A4** | **A5** | **Mean** | **SD** | **SD/Mean** | **SD of mean** | **Real A** | **Real/Mean** | **Points** |
| --- | --- | --- | --- | --- | --- | --- | --- | --- | --- | --- | --- | --- |
| circle | 1633.7 | 1649.0 | 1618.8 | 1642.2 | 1623.3 | 1633.4 | 12.6 | 0.008 | 5.6 | 326.7 | 1.04 | 30 |
| star | 1498.5 | 1505.3 | 1497.8 | 1504.3 | 1513.2 | 1503.8 | 6.2 | 0.004 | 2.8 | 300.8 | 2.30 | 32 |
| irregular | 2195.3 | 2215.1 | 2177.2 | 2207.8 | 2186.2 | 2196.3 | 15.4 | 0.007 | 6.9 | 439.3 | 2.18 | 25 |
|  |  |  |  |  |  |  |  |  |  |  |  |  |
| FMTI | 445.7 | 401.1 | 365.1 | 325.1 | 341.8 | 375.8 | 48.4 | 0.129 | 21.6 |  |  | 35 |
| IF | 85.1 | 39.8 | 43.8 | 47.7 | 80.4 | 59.4 | 21.6 | 0.363 | 9.6 |  |  | 20 |
| IT2 | 83.4 | 50.8 | 62.9 | 49.5 | 49.6 | 59.2 | 14.6 | 0.247 | 6.5 |  |  | 15 |

**Figure S1.** Known geometry (circle, star, irregular) used in tests of accuracy of digitisation methodology.


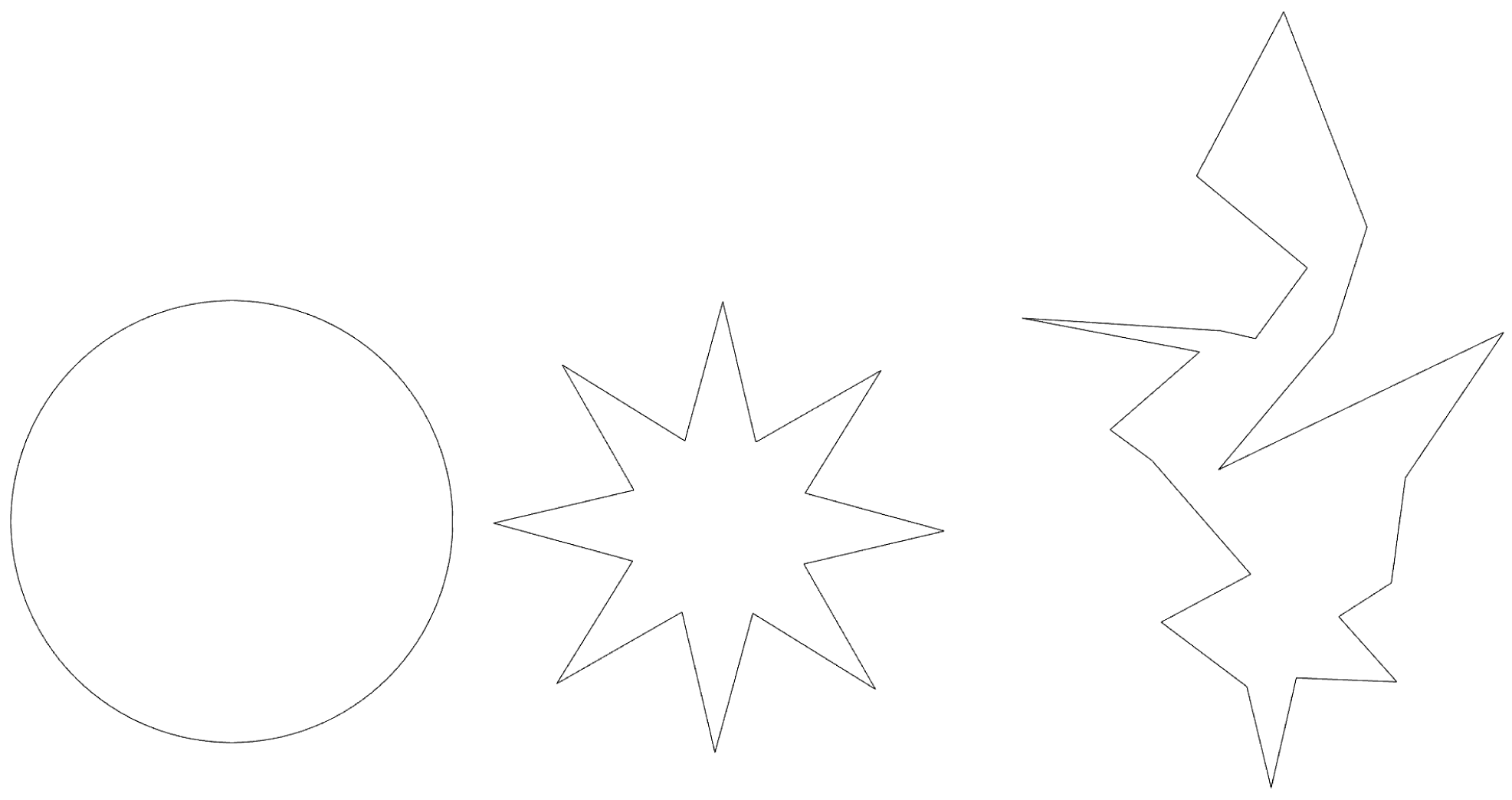


**Figure S2.** Linear regression results for predicting muscle PCSA from AA, showing (A) all data, then (B) with outliers removed, then (C) the ‘one size fits all’ regression (equation 3) used for muscles that did not have unique equations. All data originally were logged to normalise them and reduce skewness.


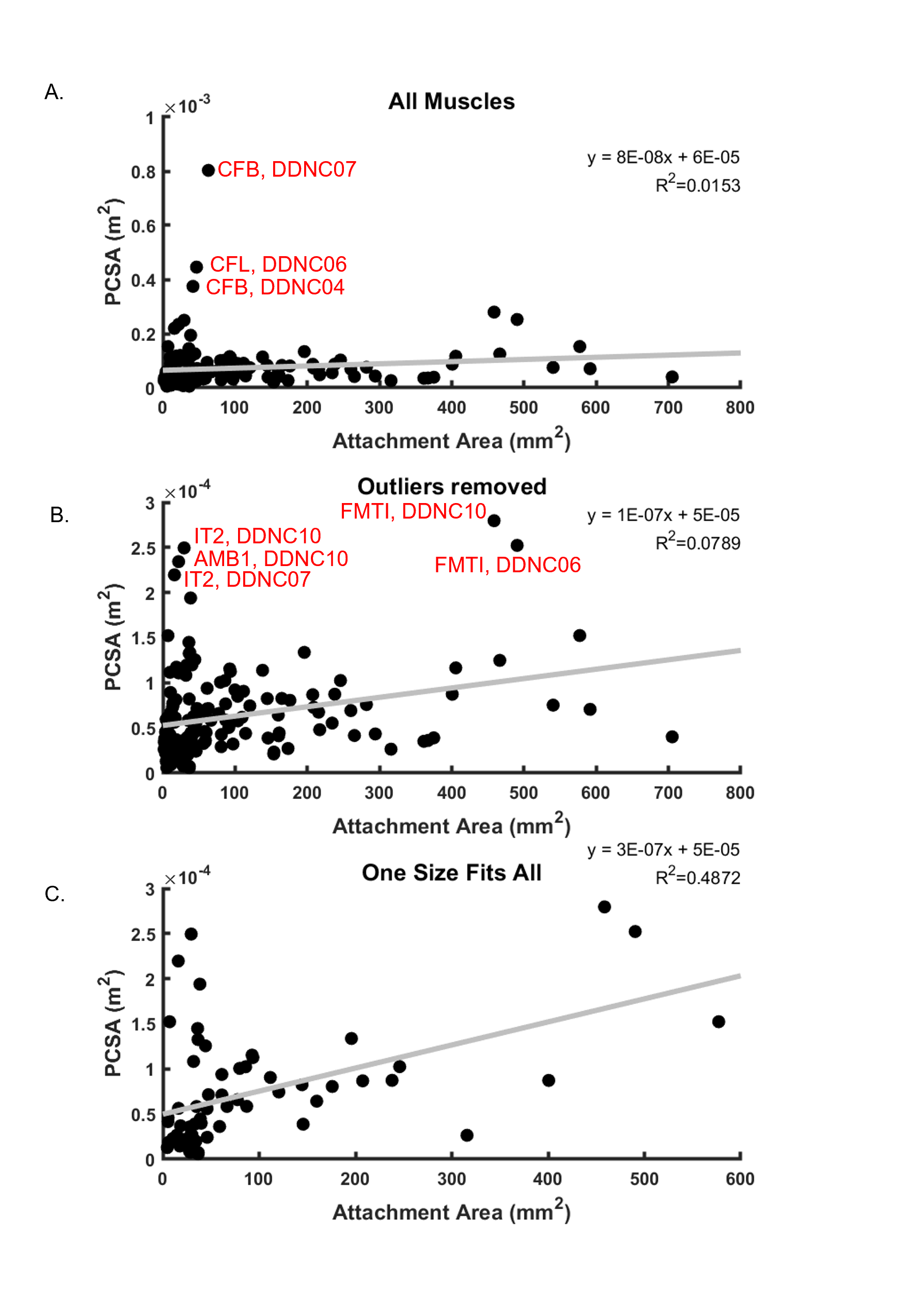

Supplement: Supplementary file 1 — Appendix S1 [file JOA-242-289-s001.docx]
